# The imprinted gene *Pw1/Peg3* regulates skeletal muscle growth, satellite cell metabolic state, and self-renewal

**DOI:** 10.1038/s41598-018-32941-x

**Published:** 2018-10-02

**Authors:** Rosa Maria Correra, David Ollitrault, Mariana Valente, Alessia Mazzola, Bjorn T. Adalsteinsson, Anne C. Ferguson-Smith, Giovanna Marazzi, David A. Sassoon

**Affiliations:** 1 UMR S 1166 INSERM (Stem Cells and Regenerative Medicine Team), University of Pierre and Marie Curie Paris VI, Paris, 75634 France; 2 Institute of Cardiometabolism and Nutrition (ICAN), Paris, 75013 France; 3 Department of Physiology Development and Neuroscience, Downing Street, University of Cambridge, Cambridge, United Kingdom; 4 Department of Genetics, University of Cambridge, Downing Street, Cambridge, United Kingdom; 5 Present Address: Institut National de la Santé et de la Recherche Médicale (INSERM), Unit 970, Paris Cardiovascular Research Center, Université René Descartes Paris, Paris, France

## Abstract

*Pw1/Peg3* is an imprinted gene expressed from the paternally inherited allele. Several imprinted genes, including *Pw1/Peg3*, have been shown to regulate overall body size and play a role in adult stem cells. *Pw1/Peg3* is expressed in muscle stem cells (satellite cells) as well as a progenitor subset of muscle interstitial cells (PICs) in adult skeletal muscle. We therefore examined the impact of loss-of-function of *Pw1/Peg3* during skeletal muscle growth and in muscle stem cell behavior. We found that constitutive loss of *Pw1/Peg3* function leads to a reduced muscle mass and myofiber number. In newborn mice, the reduction in fiber number is increased in homozygous mutants as compared to the deletion of only the paternal *Pw1/Peg3* allele, indicating that the maternal allele is developmentally functional. Constitutive and a satellite cell-specific deletion of *Pw1/Peg3*, revealed impaired muscle regeneration and a reduced capacity of satellite cells for self-renewal. RNA sequencing analyses revealed a deregulation of genes that control mitochondrial function. Consistent with these observations, *Pw1/Peg3* mutant satellite cells displayed increased mitochondrial activity coupled with accelerated proliferation and differentiation. Our data show that *Pw1/Peg3* regulates muscle fiber number determination during fetal development in a gene-dosage manner and regulates satellite cell metabolism in the adult.

## Introduction

Genomic imprinting is a mammalian-specific form of gene regulation in which one allele is repressed depending upon parental origin^1^. Although about 100–200 parentally imprinted genes have been identified to date, it remains unclear how parental imprinting contributes to gene function and how this form of epigenetic regulation was evolutionarily selected^1,2^. In addition, during development, loss or ‘relaxation’ of imprinting in specific tissue and cell types leads to bi-allelic expression of imprinted genes^3–6^. This absence of imprinting regulates specific biological processes such as the generation and maintenance of the postnatal neural stem cell pool^4,7^. Furthermore, the regulation of imprinting is proposed to maintain gene dosage in central nervous system (CNS) stem cells during development and adult life^8^.

*Pw1/Peg3* was isolated from a screen designed to identify genes that regulate skeletal muscle lineage commitment^9^, as well as being discovered an imprinted gene expressed primarily from the paternal allele^10^. During embryogenesis, *Pw1/Peg3* is expressed at high levels upon gastrulation and down-regulated during fetal and postnatal development^9^. In addition to its expression during development, we found that *Pw1/Peg3* is expressed in adult stem cells in all tissues examined thus far including skeletal muscle, skin, blood and CNS^11^. In adult skeletal muscle, *Pw1/Peg3* is expressed in satellite cells, which give rise to new muscle fibers during regeneration, as well as in a subpopulation of interstitial progenitor cells (PICs) that consist of several non-muscle progenitor lineages^12,13^.

Several *Pw1/Peg3* mutant mouse lines have been generated, including a recent line generated by our laboratory. While some differences in phenotypes have been described, all the mice share a defect in postnatal growth^14–18^. It has previously been shown that loss of *Pw1/Peg3* function results in reduced postnatal growth with a decrease in lean mass and a concomitant increase in body fat^17^. This work highlights a central role for *Pw1/Peg3* in regulating body metabolic pathways, consistent with the emerging role of imprinted genes as key players in mammalian metabolism^19^. Previous reports demonstrate that PW1 regulates two key cell stress pathways via interactions with the TNF receptor-associated factor2 (TRAF2) and p53-mediated cell death. By direct interaction with Siah1 (Seven in absentia homolog 1) and BAX (Bcl2-associate X) proteins, PW1 participates in cell death and growth arrest^20–22^. In addition, *Pw1/Peg3* has been described as a tumor suppressor in glioma cell lines and human ovarian cancer^23,24^. Moreover, we note that PW1 contains 12 Krüppel-like DNA binding zinc fingers^9,10^ and chromosomal immunoprecipitation assays reveal that a large number of its potential gene targets are involved in mitochondrial function, suggesting a link between *Pw1/Peg3* function and cell metabolism^25^. To support this hypothesis other studies have shown that *Pw1/Peg3* regulates genes involved in lipid metabolism and plays a central role in catabolic processes^15,26,27^. Together, these studies suggest that *Pw1/Peg3* controls not only whole body metabolic pathways but also the metabolic state of the cell.

Here, we investigated the role of *Pw1/Peg3* specifically in skeletal muscle including postnatal growth and adult muscle progenitor function. We used a mutant floxed allele for *Pw1/Peg3* (referred to henceforth as *Pw1*), that recombines exons 8 and 9 removing >90% of the coding domain. This mouse line was used to generate both a constitutive *Pw*1 loss-of-function mouse^18^ and to delete *Pw1* function specifically in muscle satellite cells.

We report here that *Pw1* mutant mice exhibit a decrease in myofiber number as compared to wildtype and this difference is established at birth. Interestingly, we observed that the *Pw1* maternal inherited allele is expressed at very low levels, and its loss alone has no detectable phenotype. However, deletion of both *Pw1* alleles in homozygotes has a more profound effect on myofiber number when compared to the deletion of only the paternal allele, revealing a functional contribution for maternally-inherited *Pw1* when the paternal allele is deleted. In addition to a role in fiber number determination, we found that *Pw1* deletion leads to a decline in satellite cell number and disrupts the balance between self-renewal and differentiation following injury. Transcriptome analyses comparing mutant and wildtype satellite cells reveals a down-regulation of gene expression involved in cell death and mitochondrial organization. Consistent with this, we observe that mutant satellite cells display an increase in mitochondrial activity and exit the quiescent state more rapidly than wildtype cells. Our study shows that *Pw1* gene dosage regulates skeletal muscle growth and loss of *Pw1* function abrogates satellite cell renewal and proper mitochondrial function. These findings provide further insights into the importance of imprinted genes in muscle development and homeostasis, and represent another example of selective biallelic expression of an imprinted gene in an adult stem cell niche.

## Results

### *Pw1* gene-dosage regulates skeletal muscle mass and fiber number

**Figure 1:**
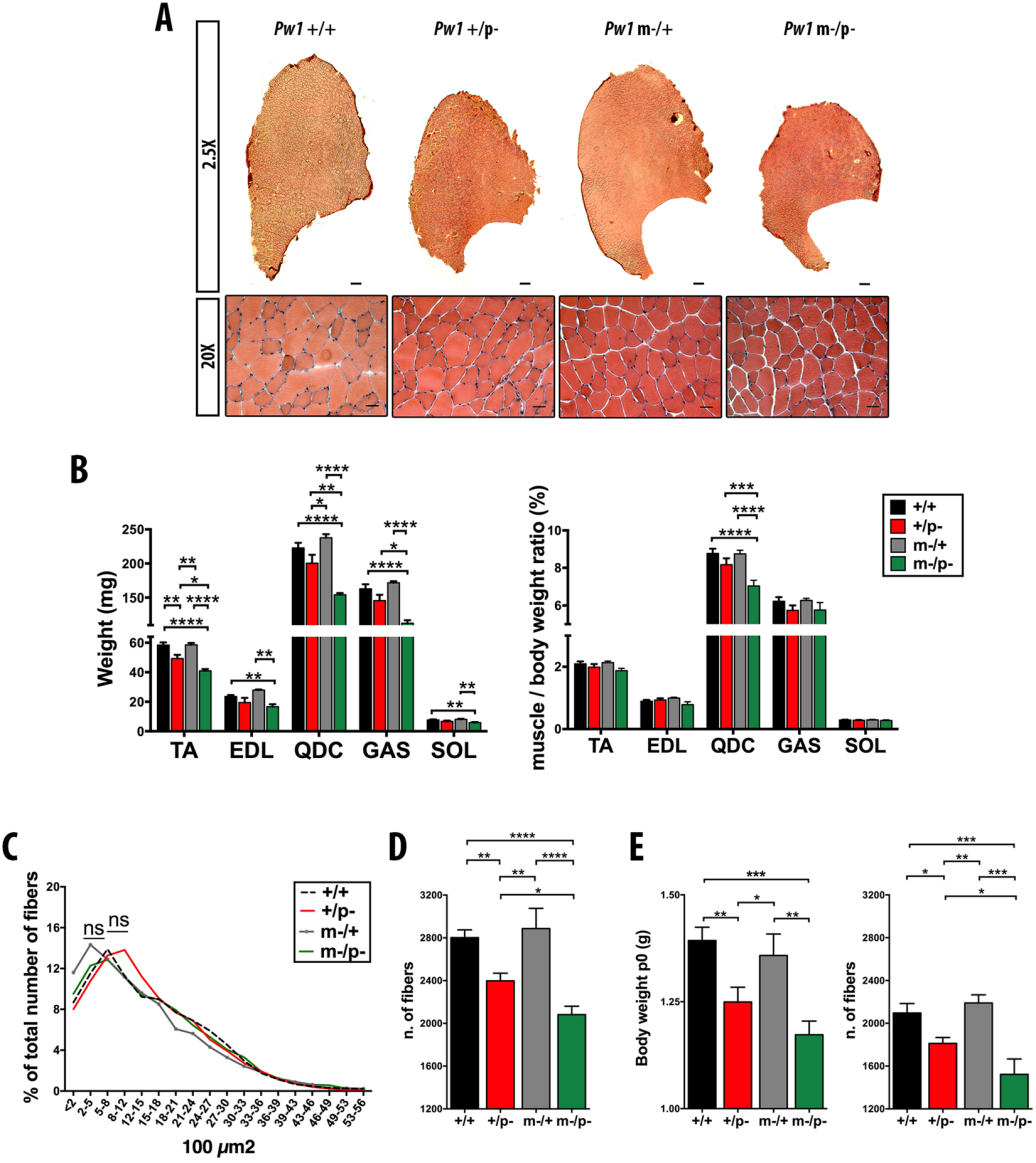
*Pw1* is required for the establishment of muscle size and myofiber number. **(A)** Representative photomicrographs at lower (upper panel; scale bars = 200 μm) and higher magnification (lower panel; scale bars = 50 μm) of *TA* muscles cross-sections from 3 month *Pw1*^+/+^, *Pw1*^+/p−^, *Pw1*^m−/+^ and *Pw1*^m−/p−^ mice stained with hematoxylin and eosin. **(B)** Histograms showing the muscle weight (left panel) and muscle/body weight ratio (right panel) of *Tibialis Anterior* (*TA*), *Extensor Digitorum Longus* (*EDL*), *Quadriceps* (*QDC*), *Gastrocnemius* (*GAS*), *Soleus* (*SOL*) muscles from 3 month *Pw1*^+/+^, *Pw1*^+/p−^, *Pw1*^m−/+^ and *Pw1*^m−/p−^ mice (n = 10 for each genotype). *Pw1*^+/p−^ and *Pw1*^m−/p−^ muscles are smaller than *Pw1*^+/+^ and *Pw1*^m−/+^ muscles. *Pw1*^m−/p−^ *TA*, *QDC* and GAS muscles are smaller than *Pw1*^+/p−^ *TA*, *QDC* and *GAS* muscles. **(C)** Fiber size distribution in 3 month *Pw1*^+/+^ (dash), *Pw1*^+/p−^ (red), *Pw1*^m−/+^ (grey) and *Pw1*^m−/p−^ (green) *TA*. Values represent the mean number ± s.e.m. per 100 fibers (n = 5 for each genotype). Statistical analyses were performed using Student’s t-test. *Pw1* depletion did not result in fibers area differences. **(D)** Histograms representing the fiber number in 3 month *Pw1*^+/+^, *Pw1*^+/p−^, *Pw1*^m−/+^ and *Pw1*^m−/p−^ *TA* muscles sectioned through the midbelly region (n = 7 for each genotype). The number of fibers is decreased in *Pw1*^+/p−^ and *Pw1*^m−/p−^ *TA* muscles as compared to *Pw1*^+/+^ and *Pw1*^m−/+^ *TA* muscles. *Pw1*^m−/p−^ *TA* muscles have reduced number of muscle fibers as compared to *Pw1*^+/p−^. **(E)** Left panel-Histograms showing total body weight of postnatal day 0 (P0) *Pw1*^+/+^, *Pw1*^+/p−^, *Pw1*^m−/+^, and *Pw1*^m−/p−^ mice (n = 14 for each genotype). *Pw1*^+/p−^ and *Pw1*^m−/p−^ are smaller as compared to *Pw1*^+/+^ and *Pw1*^m−/+^ mice. Right panel-Histograms representing the fibers number in P0 *Pw1*^+/+^, *Pw1*^+/p−^, *Pw1*^m−/+^, and *Pw1*^m−/p−^ mice *TA* muscles (n = 5 for each genotype). *Pw1*^+/p−^ and *Pw1*^m−/p−^ *TA* have less fiber as compared to *Pw1*^+/+^. *Pw1*^m−/p−^ *TA* displays less number of fibers as compared to *Pw1*^+/p−^ *TA*. Values are expressed as mean ± s.e.m. Statistical analyses were performed using one-way ANOVA and Tukey’s post-test for multiple comparison *P < 0.05, **P < 0.01 and ***P < 0.001 (n ≥ 4); n.s., not significant. In all the graphs *Pw1*^+*/*+^ (+/+), *Pw1*^+/p−^ (+/p−), *Pw1*^m−/+^ (m−/+), and *Pw1*^m−/p−^ (m−/p−).

Skeletal muscle represents ~50% of total body mass, therefore we investigated whether the decrease in lean mass and overall body size of *Pw1* mutant mice was due to changes in muscle tissue. Hind limb skeletal muscles from all 4 genotypes (wild type, *Pw1*^+/+^; heterozygotes with the paternally inherited allele deleted, *Pw1*^+/p−^; heterozygotes with the maternally inherited allele deleted, *Pw1*^m−/+^; homozygotes for the mutant allele, *Pw1*^m−/p−^) of 3 month old male mice were examined. *Pw1*^+/p−^ and *Pw1*^m−/p−^ muscles displayed an overall reduction in weight and cross-sectional area as compared to wildtype (*Pw1*^+/+^), whereas no differences were detected between *Pw1*^m−/+^ and wildtype mice (Fig. 1A,B). In particular, the larger limb muscles, such as the *Tibialis Anterior*, *Quadriceps* and *Gastrocnemius*, were significantly reduced in size and mass in *Pw1*^m−/p−^ mice as compared to the same muscle from *Pw1*^+/p−^ mice, revealing a contribution of the *Pw1* maternal allele to muscle growth in the absence of the canonically expressed paternally inherited copy (Fig. 1A,B). Since the overall body size and weight of *Pw1* mutant mice are decreased^18^, we normalized muscle mass to total body weight and observed that, with the exception of the *Quadriceps*, muscle mass was reduced proportionally to body mass. We next measured myofiber cross-sectional area (CSA) and fiber number in the *Tibialis Anterior* (*TA*) muscle. *Pw1*^+/p−^ and *Pw1*^m−/p−^ fiber size distributions were unaffected as compared to wildtype and *Pw1*^m−/+^ (Fig. 1A,C). In contrast, the total number of *TA* fibers was significantly lower in the *Pw1*^+/p−^ and *Pw1*^m−/p−^ mice, when compared to the wildtype (Fig. 1D). The decrease in myofiber number was not accompanied by any change in relative distribution of fiber types (*Type2A*, *2B* and *1* fibers) (Fig. S1A). Moreover, the reduction in fiber number was more pronounced in mice lacking both the paternal and maternal alleles (*Pw1*^m−/p−^) than when only the paternal allele was deleted (*Pw1*^+/p−^) (Fig. 1D), further supporting a role for the maternal allele in this process. Taken together, these results show that both *Pw1* alleles can participate in the establishment of muscle fiber number whereas fiber type and size are unaffected.

The mechanisms underlying myofiber number determination are not fully elucidated, however several studies suggest that skeletal muscle fiber number is determined during embryonic/fetal development^28,29^. Comparison of the body weight of newborn (P0) mice in all four genotypes indicated that *Pw1*^+/p−^ and *Pw1*^m−/p−^ mice display a slightly reduced weight compared to wildtype (Fig. 1E-left panel). We quantified total myofiber number in newborn *TA*s from all four genotypes. The fiber number was significantly lower in the *TA* from *Pw1*^+/p−^ and *Pw1*^m−/p−^ mice as compared to *Pw1*^m−/+^ and *Pw1*^+/+^mice (Fig. 1E-right panel). Additionally, newborn *Pw1*^m−/p−^ mice displayed a higher reduction on the fiber number as compared to *Pw1*^+/p−^. Taken together, these results show that *Pw1* plays a role during fetal development in the determination of muscle fiber number and while loss of the maternal allele alone has no observable phenotype, the loss of both alleles results in a stronger phenotype demonstrating a contribution of the maternal allele to the establishment of myofiber number.

### *Pw1* is transcribed from the maternal allele in skeletal muscle

**Figure 2:**
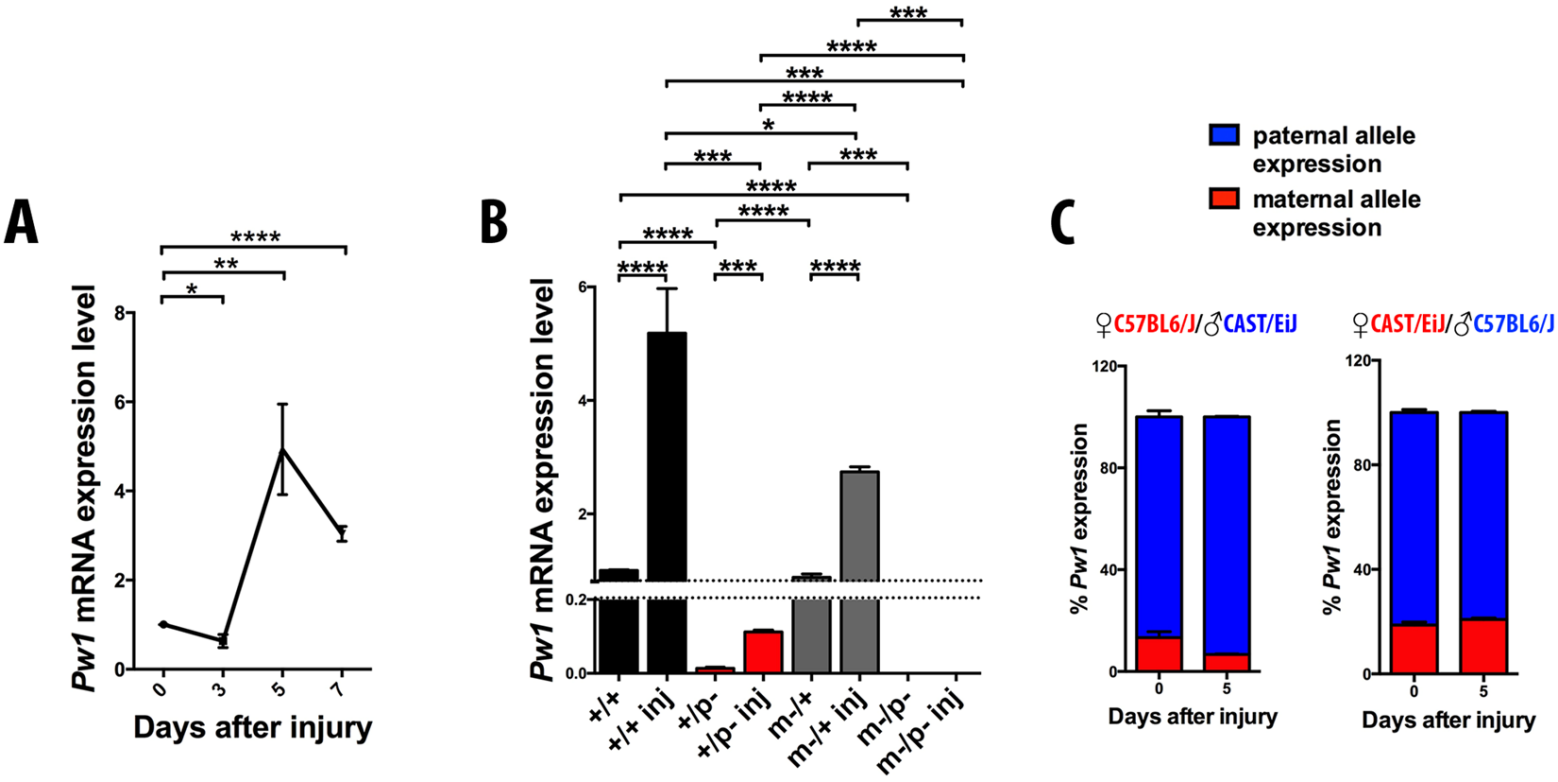
Paternal and maternal *Pw1* alleles are transcribed in skeletal muscle. (**A)** Expression levels of *Pw1* wildtype allele normalized to *Hprt1* gene expression from real time PCR at three, five, and seven days after CTX injury in adult *Pw1*^+/+^ *TA* muscle (n = 3 for each genotype). *Pw1* has a peak of expression five days after muscle injury. **(B)** Expression levels of *Pw1* wildtype allele from real time PCR normalized to *Hprt1* gene expression in uninjured and five days after injury adult *Pw1*^+*/*+^, *Pw1*^+/p−^, *Pw1*^m−/+^, and *Pw1*^m−/p−^ *TA* (n = 3 for each genotype). Maternal *Pw1* transcript is detected in injured *Pw1*^+/p−^ muscles. **(C)** *Pw1* allele specific expression in adult *TA* muscle before and five days after CTX injury from reciprocal hybrid offspring of C57BL6/J and CAST/EiJ mice (n = 3 for each genotype). Bi-allelic *Pw1* expression was observed in adult injured and uninjured muscle tissue. Values are expressed as mean ± s.e.m. In all the graphs statistical analyses were performed using Student’s t-test *P < 0.05, **P < 0.01 and ***P < 0.001. In all the graphs *Pw1*^+*/*+^ (+/+), *Pw1*^+/p−^ (+/p−), *Pw1*^m−/+^ (m−/+), and *Pw1*^m−/p−^ (m−/p−).

*Pw1* is considered to be expressed from the paternal allele, however the observation that the homozygous mutant (*Pw1*^m−/p−^) has a stronger fiber number phenotype as compared to the loss of only the paternal allele (*Pw1*^+/p−^), suggests that the maternally inherited allele has a required function. We showed previously that neonatal muscle expresses higher levels of *Pw1* as compared to the adult^9,20^, however expression levels are elevated in the adult in response to injury with a peak of expression occurring five days after muscle damage (Fig. 2A).

In order to assess the absence or presence of maternal *Pw1* transcripts in skeletal muscle, we used real time PCR to detect the *Pw1* wildtype allele in adult uninjured and injured skeletal muscle from 3 month *Pw1*^+*/*+^, *Pw1*^+/p−^, *Pw1*^m−/+^, and *Pw1*^m−/p−^ *TA*. Using primers specific for the *Pw1* wildtype allele, we observed maternal *Pw1* transcripts in adult uninjured as well as 5 days following cardiotoxin (CTX) injury in *Pw1*^+/p−^ muscle (Fig. 2B). We next stained muscle tissue sections from *Pw1*^+/+^, *Pw1*^+/p−^, *Pw1*^m−/+^ and *Pw1*^m−/p−^ newborn muscle for PW1 expression but did not observe detectable levels of PW1 protein in either *Pw1*^+/p−^ or *Pw1*^m−/p−^ muscles samples as compared to *Pw1*^m−/+^ and *Pw1*^+/+^ muscle section (Fig. S1B). These results suggest that the levels of PW1 protein are either below detection limits or that the maternal transcript is not translated. Taken together, these data show an activation of the *Pw1* paternal allele upon injury and, in the absence of an intact paternal allele, the maternal copy is also activated.

To determine whether the maternal expression observed was also evident in the presence of an intact paternally inherited *Pw1* allele, we assessed allele-specific *Pw1* expression in muscle tissue before and after injury in hybrid offspring from reciprocal crosses of *Mus musculus domesticus* (C57BL6/J) and *Mus musculus castaneus* (CAST/EiJ) strains. As a negative control (background), we measured the expression of CAST/EiJ *Pw1* allele transcript from C57BL6/J X C57BL6/J crosses and vice versa in adult muscle tissue before and after injury (Fig. S2A–C). A comparable significant increase above background was quantified between pre- and post-injury states (Fig. S2B,C). These data show that in wild type animals, *Pw1* transcription derives predominantly from the paternally inherited allele but that there is a low level of transcription from the maternal allele (5–15% depending on genetic background) (Fig. 2C). Importantly however, activation of the maternal allele was not observed after injury in the presence of an intact paternally inherited *Pw1*. Hence, maternal allelic activation upon injury is only evident in *Pw1* mutants.

In adult skeletal muscle, *Pw1* expression is restricted to two progenitor populations: satellite cells and PICs (PW1+ interstitial cells)^12,30^. Therefore, we asked whether the *Pw1* maternal transcript distribution is cell-type specific. Using real time PCR, we analyzed *Pw1* wildtype allele expression in satellite cells and the fibro adipogenic progenitor (FAPs), which represent a subpopulation of PICs, isolated from *Pw1*^+*/*+^ and *Pw1*^+/p−^ adult muscle and observed that both stem cell populations display maternal *Pw1* transcript expression (Fig. S3A). Taken together, these results reveal that the maternal *Pw1* transcript is constitutively expressed at low levels in skeletal muscle throughout postnatal life, at least, and while overall levels increase in response to injury, the maternal transcript remains low and may not be translated.

### *Pw1* mutant satellite cells display impaired self-renewal

Satellite cells are required for proper regeneration^31,32^. As satellite cells express *Pw1*, we tested muscle regeneration in wildtype and *Pw1*^m−/p−^ mice. The *TA* muscles were injured using cardiotoxin (CTX) and examined two weeks later. We observed no overt differences between mutant and wildtype muscles, nor did we observe significant levels of fibrosis or fat infiltration, which are features of muscle regenerative defects (Fig. S4A). In addition, myofiber size (cross-sectional area, CSA) post-regeneration, was unaffected by the loss of *Pw1* function (Fig. S4B).

**Figure 3:**
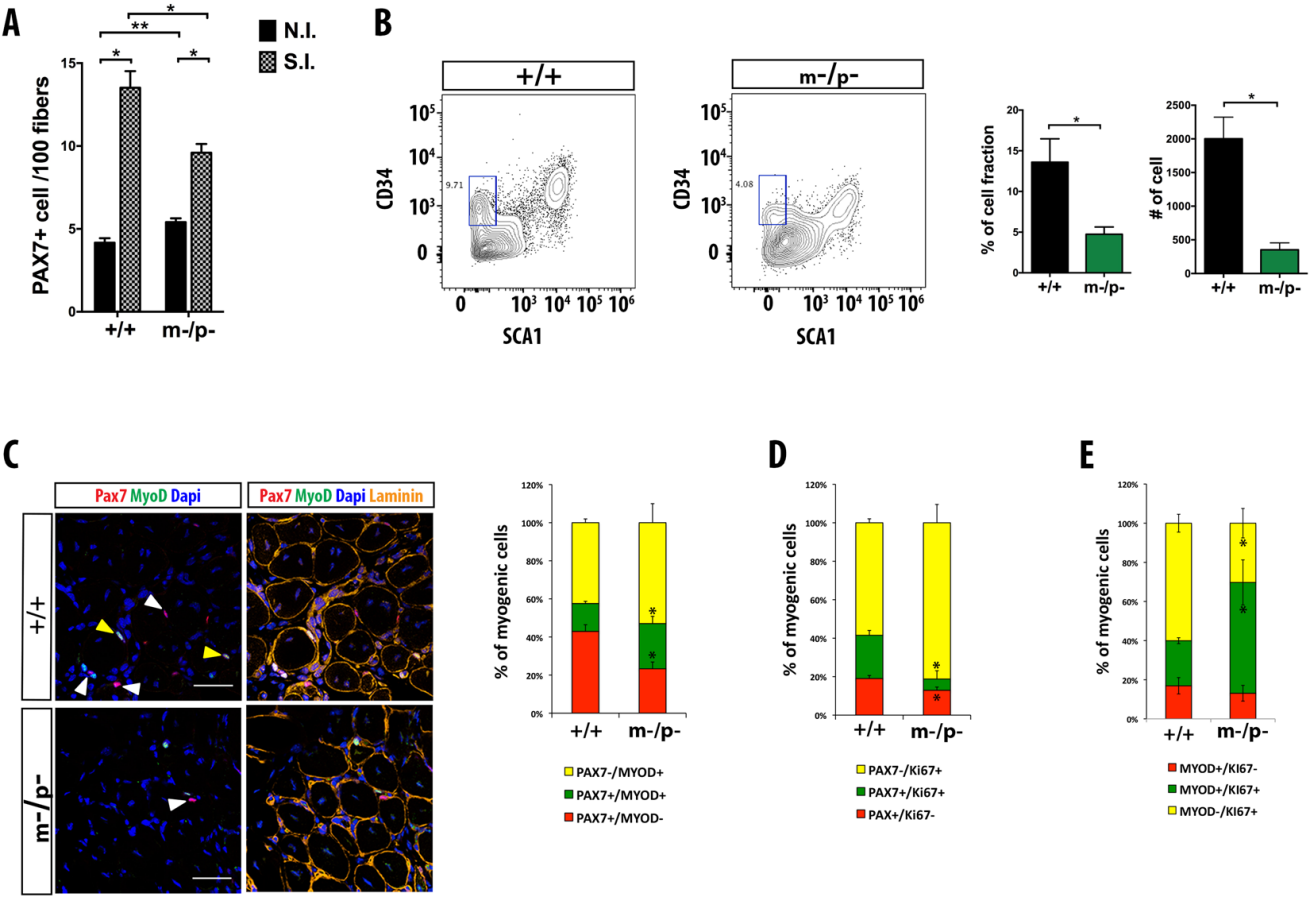
*Pw1* null satellite cells display altered self-renewal properties. **(A)** Quantification of PAX7 positive cells in *Pw1*^+/+^ and *Pw1*^m−/p−^ 3 month *TA* muscle before and after two weeks of a single CTX injury (n = 4 for each genotype). NI = non injured; SI = single injured. Values represent the mean number of positive cells ± s.e.m. per 100 fibers. The number of PAX7 positive cells is higher in uninjured *Pw1*^m−/p−^ muscle as compared to *Pw1*^+/+^. In contrast, the number of PAX7 positive cells in injured *Pw1*^m−/p−^ muscle is significantly lower as compared to *Pw1*^+/+^ two weeks after injury. (**B**) Left Panel: Flow cytometric analyses of single cells from *Pw1*^+/+^ and *Pw1*^m−/p−^ mice *TA* muscles five days after injury gated for α7-integrin^+^/CD34^+^/TER119^−^/CD45^−^/SCA1^−^. The gate used to identify CD34^+^/SCA^−^ quiescent satellite cells is shown in blue. Right Panel: Histograms showing percentages (left) and absolute cell numbers (right) of quiescent satellite cells CD34^+^/SCA^−^ fraction analyzed as shown in (left panel) (n = 3 for each genotype). Quiescent satellite cells CD34^+^/SCA^−^ population is reduced in *Pw1*^m−/p−^ injured muscle as compared to *Pw1*^+/+^. (**C**) Left Panel: Representative cross-sections of *Pw1*^+/+^ and *Pw1*^m−/p−^ *TA* muscles one week after two consecutive CTX injury immunostained for PAX7 (red), MYOD (green), and LAMININ (orange). Nuclei were visualized by DAPI. White and yellow arrows denote PAX7+/MYOD− (self-renewing) and PAX7+/MYOD+ (proliferating) myogenic cells respectively. Scale bar = 50 μm. Right Panel: Histograms showing proportions of myogenic cells PAX7+/MYOD− (red), PAX7+/MYOD+ (green) and PAX7−/MYOD+ (yellow). The proportion of self-renewing cells is decreased in *Pw1*^m−/p−^ compared *Pw1*^+/+^, whereas the proliferating and committed myoblasts show a concomitant increase (n = 3 for each genotype). (**D**) Histograms showing proportions of PAX7+/KI67− cells (quiescent satellite cells, red), PAX7+/KI67+ cells (proliferating satellite cells, green) and PAX7−/KI67+ cells (proliferating myoblast, yellow) from *Pw1*^+/+^ and *Pw1*^m−/p−^ *TA* muscles one week after two consecutive CTX injury (n = 3 for each genotype). The proportion of quiescent satellite cells is decreased in *Pw1*^m−/p−^ compared *Pw1*^+/+^. (**E**) Histograms showing proportions of myogenic cells MYOD+/KI67− (committed cells, red), MYOD+/KI67+ (proliferating and committed cells, green) and MYOD−/KI67+ (proliferating cells, yellow) from *Pw1*^+/+^ and *Pw1*^m−/p−^ *TA* muscles one week after two consecutive CTX injury (n = 3 for each genotype). The proportion of proliferating and committed cells is increased in *Pw1*^m−/p−^ compared *Pw1*^+/+^. Values are expressed as mean ± s.e.m. Statistical analyses were performed using Student’s t-test *P < 0.05, **P < 0.01 and ***P < 0.001. In all the graphs *Pw1*^+*/*+^ (+/+) and *Pw1*^m−/p−^ (m−/p−).

We next investigated the number of satellite cells based upon the expression of PAX7 in wildtype and mutant muscles before and after injury. These analyses revealed a ~10% higher number of PAX7+ cells in mutant muscle as compared to the wildtype prior to injury (Fig. 3A). We did not detect any co-staining of PAX7 and KI67 nor do we observe any staining for MYOD in *Pw1* mutant satellite cells in uninjured muscles (data not shown) revealing that there is no overt chronic cell cycle activation in the absence of injury that could account for this increased number of satellite cells in *Pw1* mutant muscles, however we cannot rule out an accumulation of satellite cells due to a low level of cell cycling in the absence of *Pw1* function. While we detected an overall increase in the number of PAX7+ cells as compared to the steady-state, we observed a ~30% decrease in satellite cell number in the mutant muscle as compared to wildtype two weeks following CTX injury (Fig. 3A). These results suggest that loss of *Pw1* function disrupts the maintenance of the satellite cell pool following injury via a loss in self-renewal capacity. To test this, we quantified the absolute number and percentage of self-renewing satellite cells in CTX injured muscle 5 days after injury by flow cytometry (Fig. 3B). Satellite cells capable of replenishing the stem cell pool (quiescent satellite cells) after acute injury are defined based on the surface expression of α7-integrin and CD34 and the absence of TER119, CD45 and SCA1 expression (after viability dye exclusion)^33^. Consistent with a reduction in self-renewal capacity, there was a marked decrease in the satellite stem cell pool in *Pw1*^m−/p−^ as compared to *Pw1*^+/+^ muscles (Fig. 3B).

Satellite cell self-renewal can be measured by tracking the populations of PAX7−/MYOD+, PAX7+/MYOD+ and PAX7+/MYOD− cells one week following injury. This corresponds to a stage during which regeneration is ongoing, consisting of committed (PAX7−/MYOD+) and expanding satellite cell derived myoblasts that will form new myofibers (PAX7+/MYOD+), as well as a smaller population of cells that restore the satellite cell population (PAX7+/MYOD−)^34,35^. Using this approach, we observed that all three populations of cells are present in wildtype and mutant muscles after injury, however the population of self-renewing satellite cells (PAX7+/MYOD−) was markedly decreased in mutant muscle, while the number of proliferating and differentiation-committed myoblasts were markedly increased (Fig. 3C). In addition, using a marker for cell proliferation (KI67), we noted a reduced percentage of non-cycling satellite cells (PAX7+ KI67−) during muscle regeneration in the mutant muscles and an increase in the percentage of committed/proliferating (KI67+ MYOD+) cells (Fig. 3D,E). Taken together, these data reveal that *Pw1* regulates satellite cell number and self-renewal capacity.

### *Pw1* loss-of-function abrogates muscle regeneration after multiple injuries

The reduced number of satellite cells following a single muscle injury in *Pw1* mutant mice suggests that satellite cell self-renewal is compromised even though muscle regeneration appeared to be normal (Fig. S4A). Since stem cell self-renewal is essential for tissue regeneration in response to multiple injuries^36–38^, we investigated the effect of *Pw1* loss in adult muscle regeneration after two consecutive injuries with CTX (double injury). H&E staining of doubly injured *TA* muscles revealed a low level of fibrosis and fat infiltration in *Pw1* mutant muscle as compared to wildtype (Fig. S5A,B) suggesting compromised regeneration. In addition, we measured fiber cross-sectional area (CSA) after double injury and we observed a decrease of the percentage of newly formed small fibers (<800 μm) in *Pw1*^m−/p−^ as compared to *Pw1*^+/+^ muscles as well as an increase of the percentage of newly formed bigger fibers (>35000 μm) (Fig. S5C). Furthermore, we noted an increased number of centrally located nuclei per fiber in doubly injured *Pw1* mutant muscle as compared to wildtype (Fig. S5D), however fiber number was maintained in both genotypes (Fig. S5E). Lastly, we observed a reduction of PAX7+ cells in mutant muscles after two injuries (Fig. S5F) consistent with results obtained following a single injury (Fig. 3A) indicating that *Pw1* deletion leads to the exhaustion of the satellite cell pool with the consecutive impairment of muscle regeneration. The larger fiber CSA coupled with an increase in myonuclear content suggested that the reduced satellite cells pool observed after multiple injuries in *Pw1* null muscle has an enhanced differentiation capacity disrupting the normal balance between satellite cell expansion and terminal differentiation.

**Figure 4:**
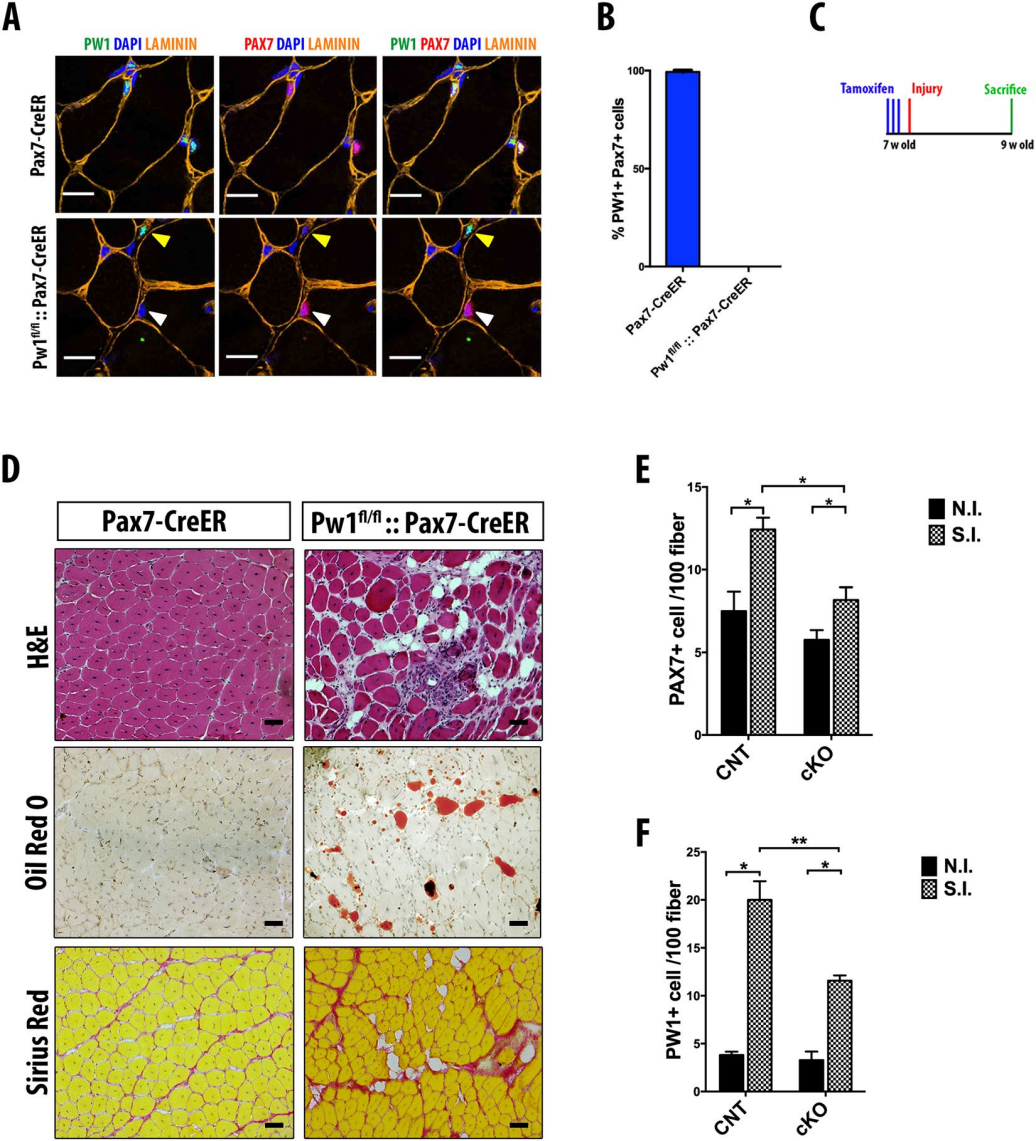
Specific *Pw1* deletion in PAX7 positive cells leads to impairment in satellite cell self-renewal. **(A)** Representative cross-section of 4 weeks-old *Pax7*-Cre and *Pax7*-Cre::*Pw1*^fl/fl^ *TA* one week after TM injection stained for PAX7 (red), PW1 (green), LAMININ (orange) and DAPI (blue). White and yellow arrows in *Pax7*-Cre::*Pw1*^fl/fl^ *TA* one week after TM injection indicate *Pw1* deletion in satellite and interstitial PW1+ cells (PICs) respectively. Scale bar = 10 µm. **(B)** Percentage of PW1+ PAX7+ cells in 4 weeks-old *Pax7*-Cre and *Pax7*-Cre::*Pw1*^fl/fl^ *TA* one week after TM injection stained as shown in (A) (n = 3 for each assay and genotype). All PAX7+ cell from *Pax7*-Cre::*Pw1*^fl/fl^ *TA* do not express PW1 as compared to *Pax7*-Cre *TA*. (**C**) Schematic representation of experimental strategy: seven weeks old *Pax7*-Cre and *Pax7*-Cre::*Pw1*^fl/fl^ mice were injected intraperitoneally with TM for three days. One day after the last TM injection, *TA* muscles were injured by a single CTX injection. Muscles were collected two weeks after injury. (**D**) Cross-sections images of *TA* muscles from *Pax7*-Cre and *Pax7*-Cre::*Pw1*^fl/fl^ mice two weeks after CTX injury stained with hematoxylin and eosin (upper panels**)**, Sirius Red (middle panels) and Oil-Red O (lower panels). Specific deletion of PW1 in satellite cells impairs skeletal muscle regeneration. (**E**,**F**) Quantification of satellite cells (PAX7+) and PICs (PW1+) per 100 fibers in *TA* from *Pax7*-Cre and *Pax7*-Cre::*Pw1*^fl/fl^ mice two weeks after CTX injury (n = 3 for each assay and genotype). Regenerating myofiber in *Pw1* depleted satellite cells have less number of satellite cells and PICs. Values represent the mean number of positive cells ± s.e.m. per 100 fibers. Statistical analyses were performed using Student’s t-test *P < 0.05, **P < 0.01 and ***P < 0.001.

In order to confirm a *Pw1* specific role in satellite cells, we used the *Pax7*-CreERT2 mice which carry a tamoxifen-inducible CRE recombinase-estrogen receptor fusion protein cassette driven by *Pax7*^31^ crossed with the *Pw1*^fl/fl^ mice^18^. The resultant *Pax7*-CRE:: *Pw1*^fl/fl^ mice were used to delete *Pw1* expression specifically in satellite cells following tamoxifen (TM) administration. Immunohistochemistry of 1 month old *Pax7*-CRE::*Pw1*^fl/fl^ and *Pax7*-CRE *TA* revealed that PW1 expression was ablated in PAX7+ cells with very high efficiency one week following TM treatment (Fig. 4A,B). We next injured *TA* muscles of TM-treated *Pax7*-CRE and *Pax7*-CRE::*Pw1*^fl/fl^ mice and analyzed muscle samples two weeks later (Fig. 4C). Interestingly, we observed that specific ablation of *Pw1* expression in satellite cells led to a more severe defect in muscle regeneration including a marked increase in ectopic fat deposition and fibrosis even following a single injury (Fig. 4D). This stronger phenotype may reflect compensatory mechanisms that are established during development and postnatal life in the constitutive *Pw1*^m−/p−^ mouse model as compared to the conditional *Pax7*-CRE::*Pw1*^fl/fl^ targeted specifically to satellite cells. We note that as *Pw1* is also expressed in the interstitial muscle cell population, these cells may participate in this compensation. Furthermore, as the Cre protein is expressed in the conditional allele in a genetic background in which one of the *Pax7* alleles is recombined, there may be an additive effect that is not seen when the *Pw1* allele is not recombined. Consistent with our previous results, we observed a decline in PAX7+ cells following injury in *Pax7*-CRE:: *Pw1*^fl/fl^ (cKO) as compared to *Pax7*-CRE (CNT) (Fig. 4E). While the *Pax7*-CRE model leads to a loss of *Pw1* expression in satellite cells, we noted that the PW1+ PAX7− interstitial cells (PICs) were also reduced in number (Fig. 4F), suggesting that loss of *Pw1* function in satellite cells has an effect on neighboring niche populations.

### *Pw1* regulates mitochondrial function metabolically primes satellite cell activation

**Figure 5:**
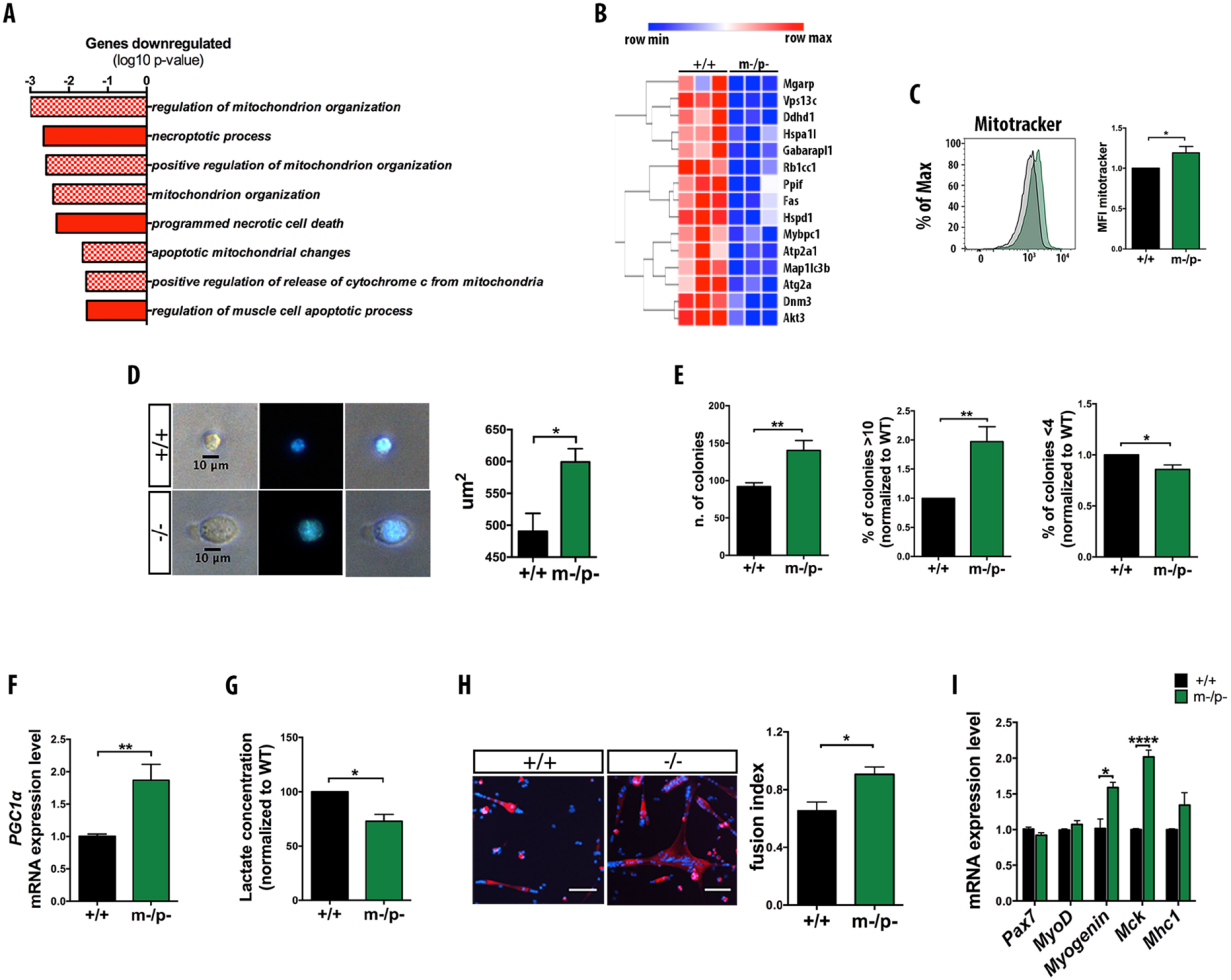
*Pw1* deletion results in satellite cell activation. (**A**) Gene Ontology (GO) analysis of downregulated genes in RNA-seq analysis of *Pw1*^m−/p−^ satellite cell. GO term represent the biological processes, dashed histograms indicates genes involved in mitochondrial activity and red histograms denote genes implicated in cell death. The x-axis shows the *p*-value (−log_10_). (**B)** GO clustering analysis for mitochondrial organization gene categories. Data were obtained from RNA-seq analysis of FACS sorted satellite cells from *Pw1*^+*/*+^ and *Pw1*^m−/p−^ mice. The gene tree is shown on the left and gene coloring was based on normalized signals as shown. Each row represents one gene, with red representing high expression and blue representing low expression (n = 3 for each genotype). (**C**) Left panel: Representative FACS analysis of Mitotracker fluorescence in *Pw1*^+/+^ (black) and *Pw1*^m−/p−^ (green) satellite cells. Right panel: Histogram represent Mitotracker mean fluorescence intensity (MFI) of *Pw1*^m−/p−^ satellite cells relative to *Pw1*^+/+^ (n = 6 for each genotype). Loss of PW1 corresponds to an increase of mitochondrial activity in satellite cell. (**D**) Left panel: Representative images of FACS isolated *Pw1*^+/+^ and *Pw1*^m−/p−^ satellite cells grown for 24 h in growth media (GM) and stained with DAPI. Right panel: Histogram showing quantitative evaluation of FACS isolated *Pw1*^+/+^ and *Pw1*^m−/p−^ satellite cells area (in square micrometers), grown for 24 h in GM (n = 3 for each genotype). *Pw1*^m−/p−^ satellite cells are larger than wildtype. (**E**) Histograms showing quantifications of the number of satellite cells per colonies (Left panel), percentages of satellite cell colonies bigger than 10 cells (Middle panel), and percentages of satellite cell colonies smaller than 4 cells (Right panel) from FACS purified satellite cells from adult (3 month) wildtype and *Pw1*^m−/p−^ muscles grown for 5 days in GM. *Pw1*^m−/p−^ satellite cells display increased proliferative capacity as compared to wildtype (n = 3 for each genotype). (**F**) Expression levels of *PGC-1α* from real time PCR normalized to *Tbp* gene expression from FACS isolated *Pw1*^+/+^ and *Pw1*^m−/p−^ satellite cells satellite cells grown for 5 days in GM (n = 3 for each genotype). *Pw1*^m−/p−^ satellite cells display increased expression level of *PGC-1α* gene as compared to wildtype. (**G**) Lactate production from FACS isolated *Pw1*^+/+^ and *Pw1*^m−/p−^ satellite cells grown for 5 days in GM (n = 4 for each genotype). *Pw1*^m−/p−^ satellite cells display decrease secretion of lactate as compared to wildtype. (**H**) Left panel: Representative images of FACS isolated *Pw1*^+/+^ and *Pw1*^m−/p−^ satellite cells immunostained for MHC (red) and counterstained with DAPI (blue). Satellite cells were grown for 48 h in differentiation media (DM). Scale bar = 200 µm. Right panel: Fusion index (mean percentage of nuclei incorporated into MHC+ cells) of differentiating wildtype and *Pw1*^m−/p−^ satellite cells stained as shown in left panel. *Pw1*^m−/p−^ differentiated satellite cells show increased Myogenic differentiation (n = 4 for each genotype). (**I**) Expression levels of *Pw1*, *Pax7*, *MyoD*, *Myogenin*, *Mck* and *Mhc1* from real time PCR normalized to *Tbp* gene expression in differentiated FACS isolated *Pw1*^+/+^ and *Pw1*^m−/p−^ satellite cells. Differentiating *Pw1*^m−/p−^ satellite cells display increased expression level of *Myogenin* and *Mck* gene as compared to wildtype (n = 3 for each genotype). Value are expressed as mean ± s.e.m. In all the graphs statistical analyses were performed using Student’s t-test *P < 0.05, **P < 0.01 and ***P < 0.001. In all the graphs *Pw1*^+*/*+^ (+/+) and *Pw1*^m−/p−^ (m−/p−).

To elucidate how *Pw1* affects satellite cell function, transcriptome analyses was performed on satellite cells from *Pw1*^+*/*+^ and *Pw1*^m−/p−^ mice by RNA-sequencing (RNA-seq) (Table S1). Satellite cells were freshly isolated from whole hind-limb muscles of 3 months mice. *Pw1*^m−/p−^ satellite cells displayed 211 downregulated genes (*p* < 0.05, log2 fold change <−0.5) and 208 upregulated genes (*p* < 0.05, log2 fold change >0.5) when compared to wildtype mice. Gene Ontology (GO) analyses revealed downregulation of the expression of genes related to mitochondrial organization and activity as well as cell death (Fig. 5A) (Figs 5B and S6A) (Table S2). Among the list of genes involved in mitochondrial organisation were known inhibitors of mitochondrial function, suggesting an increase in mitochodrial activity in satellite cells lacking *Pw1* expression^39–41^. Consistent with this, we observed an increase in mitochondrial membrane potential in freshly isolated mutant satellite cells as compared to wildtype (Fig. 5C) as measured by Mitotracker staining^42^.

Recent studies have described a cell cycle phase that occurs prior to G1 entry referred to as G_Alert_ defined by cellular metabolic activation^43^. Specifically, G_Alert_ entry entails an increase in mitochondrial activity and an increase in satellite cell size with entry into this phase being regulated by mTORC1 activation and its downstream targets including phospho-S6 (pS6)^43–45^. While no significant differences in the size of freshly sorted *Pw1*^+*/*+^ and *Pw1*^m−/p−^ satellite cells were observed (Fig. S6B), we detected a striking difference in size of *Pw1* mutant and wildtype satellite cells one day after plating (Fig. 5D). The percentages of PS6+ PAX7+ cells in mutant and wildtype adult uninjured muscle are similar indicating that the cells are not fully in a G_Alert_ phase as previously described, but rather are metabolically primed (Fig. S6C). Consistent with this, mutant satellite cells form more and larger colonies as compared to wildtype (Fig. 5E). It has been shown that satellite cell activation involves a switch from glycolytic to mitochondrial metabolism^46^ and that several mitochondrial genes such as *PGC-1α* regulate mitochondrial biogenesis and respiration to direct cells to oxidative metabolism^47,48^. We observed an increased in *PGC-1α* gene expression in *Pw1*^m−/p−^ proliferating satellite cells as compared to *Pw1*^+/+^ (Fig. 5F). Furthermore, a reduction in lactate production in proliferating mutant satellite cells was observed, indicating an accelerated switch from glycolysis to mitochondrial metabolism in *Pw1*^m−/p−^ satellite cells as compared to *Pw1*^+/+^ (Fig. 5G). In addition, *Pw1*^m−/p−^ satellite cells form larger myotubes as compared to wildtype (Fig. 5H) and upregulate myogenic markers such as *myogenin* and *Mck* (Fig. 5I) consistent with a higher state of cell activation and a shift towards differentiation commitment. Taken together, these data suggest that *Pw1* represses satellite cell activation and participates in maintaining a quiescent state.

## Discussion

We generated a novel conditional mutant mouse model for *Pw1*^18^ to analyze the role of *Pw1* during skeletal muscle growth and in adult muscle stem cell function. Previous studies, including our initial description of a *Pw1* mutant allele, have shown that constitutive loss of *Pw1* function results in a postnatal growth defect^14,17,18,49,50^. In this study, we show that the loss of *Pw1* causes a reduction in muscle mass accompanied by a ~30% decrease in myofiber number, but not in the myofiber cross-sectional area. Previous studies have shown that skeletal muscle fiber number is determined during embryogenesis in two waves of myogenesis (ED 11–14 and ED 14–16)^51,52^ corresponding to a stage when *Pw1* also shows a peak of expression^9^. Other imprinted genes have been implicated in modulating muscle mass and myofiber number. This includes the polar overdominance muscle hypertrophy phenotype of the *Callipyge* sheep and related phenotypes associate with the altered dosage of imprinted genes at the *Dlk1-Dio3* cluster in mice^53,54^. Loss of the maternally expressed genes *Grb10* and *H19* results in an overgrowth phenotype associated with an increase in myofiber number, whereas deletion of the paternally expressed genes *Mest* and *Dlk1* leads to a decrease in myofiber number^55–58^. In these studies and the present analysis, the precise cellular basis for myofiber number differences has not been elucidated, and likely reflects complex interaction between the developing muscle connective tissue and forming myofibers. As *Pw1* is expressed in both the muscle interstitium and myogenic cells, further analyses targeting loss-of-function in the interstitium will be required. *Odd skip related 1* (*Osr1)* has been shown recently to be expressed in the developing muscle connective tissue, as well as the emerging fibroadipogenic population that constitutes a large portion of the PICs population, and loss of *Osr1* function results in disorganized and poorly formed myofibers^59^. Thus, it is possible that the decrease in myofiber number observed in *Pw1* mutant muscle results from a loss of *Pw1* function in the muscle interstitium. Our data point to a key contributing factor in the postnatal growth defects observed in the *Pw1* mutant mice; namely, while newborn mutant and wildtype mice do not show pronounced body size differences, the observation that mutant mice possess a decreased myofiber number poses a limit on final achievable body size.

Genomic imprinting is a form of epigenetic regulation that is limited to 100–200 mammalian genes^1^ and the selective advantage of parental imprinting remains obscure. Emerging evidence suggests that genomic imprinting is a mechanism of gene dosage control that regulates body growth and stem cell function^4,7,60^. In the present study, we found that the reduction in muscle fiber number is more pronounced in mice lacking both paternal and maternal *Pw1* alleles as compared to muscle where only the paternal allele is deleted, suggesting that the maternal allele contributes to muscle growth in contexts where the paternal allele is absent. Recent studies have reported that *Pw1* undergoes a relaxation of imprinting leading to the expression of the canonically repressed maternal allele in newborn and adult brain^18,61^. We confirmed a low level of *Pw1* transcription from the maternal allele by assessing its imprinting status in the adult muscle of hybrid offspring from reciprocal crosses of *Mus musculus domesticus* (C57BL6/J) and *Mus musculus castaneus* (CAST/EiJ) strains in addition to verifying that either the wildtype maternal or the recombined maternal (truncated) allele are expressed in our heterozygous mutant mice. We note however that in mice carrying the mutant paternal allele and the wildtype maternal allele, we do not observe detectable levels of PW1 protein expression. Therefore, while our results demonstrate that *Pw1* gene dosage participates in the establishment of muscle fiber number, it is not clear whether this is a result of overall protein levels or an interaction between the two *Pw1* alleles via an undetermined mechanism. Regardless, our results, combined with the results of others^55–58^ reveal an interesting pattern in which maternally and paternally imprinted genes exert opposite effects upon muscle mass through the control of myofiber number.

Growing evidence points to a role for imprinted genes in adult stem cells^11,62–64^. *Pw1* is part of a group of imprinted genes, referred to as the “imprinted gene network” (IGN). Members of the IGN are expressed at high levels during embryonic development, and whereas the overall expression levels decline postnatally, they remain highly expressed in adult stem cells^63^. *In vivo* and *in vitro* deletion of different members of the IGN reveal key roles in adult stem cell maintenance and self-renewal^4,7,56,65,66^. We have shown that *Pw1* is specifically expressed in a wide range of adult stem cells^11^. Furthermore, it has been recently reported that *Pw1* regulates adult mesoangioblast competence and that PW1 expressing cells correspond to competent and self-renewing cells^11,67,68^. In skeletal muscle, *Pw1* expression is confined to two progenitor cell populations: satellite cells and interstitial cells (PICs)^12,13^. We observe here that *Pw1* loss of function leads to a decline in muscle regenerative capacity coupled with fat deposition and the exhaustion of the satellite cell pool. Furthermore, our data indicate that *Pw1* has a distinct role during fetal muscle development in the determination of muscle fiber number versus its role in adult satellite cells. To properly address this issue will require specific conditional alleles to the various cells types in developing skeletal muscle since the connective (interstitial) cells are known to play a role in fiber number determination and also express *Pw1* during development and in the adult^59^. We note that recent results regarding the role of *Osr1* during muscle development and in the adult reveal similarly distinct developmental roles and we further note that *Osr1* and *Pw1* expression largely overlap in the interstitial muscle cell population^12,13,30,59,69^.

Our findings raises the question regarding how *Pw1* regulates adult stem cell function, and in particular, stem cell competence and self-renewal. Satellite cells are a quiescent cell population in adult skeletal muscle that is activated in response to muscle injury^70^. Replenishment of the satellite cell population by self-renewal is pivotal for skeletal muscle homeostasis and defects in this process compromise muscle regeneration^71–73^. Accumulating evidence points to a key role for the satellite cell niche in satellite cell fate determination^73–75^. We report here that *Pw1* mutant satellite cells display an impaired self-renewal and specific mouse crosses that ablate *Pw1* expression exclusively in satellite cells have a profound impact on muscle regeneration with fat and fibrotic tissue deposition. The intramuscular fat infiltration observed in *Pw1* null regenerating muscle may result from a disruption of the muscle stem cell niche as well as satellite cell transdifferentiation along the adipocyte program^76^. These studies reveal that *Pw1* participates in the regulation of satellite cell activation and in turn, controls self-renewal capacity. RNA-seq analyses of purified satellite cells from wildtype and *Pw1* null mutant (*Pw1*^m−/p−^) mice revealed that multiple genes involved in mitochondrial organization and cell death are downregulated in the absence of *Pw1*. Our findings are consistent with a previous report showing that PW1 interacts with the mitochondrial cell death pathway^22,77,78^. These results are also consistent with previous ChIP-sequencing analyses in the adult brain that demonstrated that PW1 binds the promoters of multiple genes involved in mitochondrial function^79^. The link between *Pw1* and mitochondrial function is further supported by the *in vivo* phenotypes reported here, revealing an increase in mitochondrial activity in *Pw1* mutant satellite cells. Recent studies have uncovered a novel satellite cell cycle state in adult resting muscle characterized by elevated mitochondrial activity referred to as “G_Alert_”^43,45^. This state occurs in satellite cells that are distal to the site of injury and has been proposed to ‘prime’ satellite cells to enter the cell cycle. It should be noted that *Pw1* mutant satellite cells exhibit some, but not all, of the characteristics of G_Alert_. It is likely that there are multiple steps involved in the transition of satellite cells from a quiescent state towards cell cycle activation in response to injury and that loss of *Pw1* in the absence of injury metabolically primes satellite cells but is insufficient to enter a G_Alert_ state identical to what has been previous described. We note however that mitochondrial function and metabolic activation are critical to cell cycle activation and mitochondrial activity plays a key role during stem cell fate regulation including governing a switch from glycolytic to oxidative (mitochondrial) metabolism^80^. In the case of satellite cells, mitochondrial activity is associated with their capacity to differentiate rather than self-renew^81^. Our previous results showing that *Pw1* is expressed in all stem cells coupled with results presented in this study demonstrating that *Pw1* regulates satellite cell self-renewal coupled with an activation of mitochondrial function provide a crucial link towards understanding a more global role for *Pw1* in stem cell regulation.

Several studies have demonstrated that loss of *Pw1* function disrupts overall body metabolism including an increase in body fat and a reduction in lean mass^17^. To date, constitutive mutants have been used primarily to study the roles of parentally imprinted genes. While these studies have proven invaluable, complex traits ranging from behavior to body growth and body composition have been ascribed to be under the control of many parentally imprinted genes expressed quite widely, including *Pw1*^62^. Hence, the mutant phenotypes reported to date may not be due to the action of a single parentally imprinted gene in any one cell type or tissue, but rather could result from complex cell and tissue interactions. Comparison of constitutive and conditionally targeted mutants provides valuable insights in this regard. However, further work will be needed in order to understand whether the defects observed in the *Pw1* constitutive mutant are due or not to a specific stem cell population. Nonetheless, using a conditional allele to target disruption of *Pw1* function in a single tissue progenitor cell type we have shown that stem cell function is disrupted demonstrating a clear role for *Pw1* in postnatal stem cell function *in vivo* further illustrating the importance of imprinted genes in the regulation of the postnatal stem cell niche.

## Material And Methods

### Mice

Mouse models used were *Pw1* floxed (*Pw1*^fl/fl^) mice and constitutive *Pw1* knock-out (*Pw1*^m−/p−^)^18^. Mice were maintained on a C57BL6 background. All four genotypes, *Pw1*^+*/*+^*(wildtype)*, *Pw1*^*m*+/p−^ *(paternal deletion)*, *Pw1*^*m*−*/p*+^ (*maternal deletion*) and *Pw1*^m−/p−^ (*homozygous deletion*), were analyzed. Tg: *Pax7*Cre^ERT2^ mice^82^; Tg: *Pax7*Cre^ERT2^ mice were crossed with *Pw1* floxed (*Pw1*^fl/fl^) mice to obtain specific deletion of *Pw1* in PAX7+ cells. Approval for the animal (mouse) work performed in this study was obtained through review by the French Ministry of Education (Agreement#A751320).

### RNA extraction and real time PCR

Total RNA was extracted using RNeasy Micro Kit (Qiagen) and RNeasy Mini Kit (Qiagen) according to manufacturer guidelines. RNA was treated with RNase-free DNase I (Qiagen) to remove genomic DNA. RNA was reverse-transcribed using SuperScript III First-Strand Synthesis System (Thermo Fisher). Cycling conditions and primers were used as previously described^18^.

### Real time PCR primers

*Gabarapl1* forward CATCGTGGAGAAGGCTCCTA, *Gabarapl1* reverse ATACAGCTGGCCCATGGTAG

*Ppif* forward TGGCTCTCAGTTCTTTATCTGC, *Ppif* reverse ACATCCATGCCCTCTTTGAC

*Akt3* forward GGATCACAGATGCAGCTACC, *Akt3* reverse GTAGAAAGGCAACCTTCCACAC

*Atp2a1* forward ACACAGACCCTGTCCCTGAC, *Atp2a1* reverse TGCAGTGGAGTCTTGTCCTG

*Vps13c* forward CACAAGCATTGAAGATAGAAGCAAAA, *Vps13c* reverse AGTGATGGCACAATGTCTTGTTG

*Mgarp* forward AAAGAACAAACAAAGGCGGAGTTG, *Mgarp* reverse CACACTTGCTCGGCTTCTGC

*Hspa1l* forward AGAGTTGTGTGCAGACCTGT, *Hspa1l* reverse CCGGGTTGGTTGTCAGAGTA

### Tamoxifen treatment

7 weeks-old *Pax7*Cre^ERT2^::*Pw1*^fl/fl^ mice were injected intraperitoneally daily for 3 days with tamoxifen (TM) (150 μl, 20 mg/ml; Sigma Aldrich) diluted in sunflower seed oil/5% ethanol.

### Regeneration assays

Skeletal muscle regeneration was induced by intramuscular CTX injection (0.06 mg/ml, Sigma) and muscles were analyzed 14 days after injury. To analyze satellite cell self-renewal, muscle regeneration was induced by focal freeze crush, a second injury performed with a 15 days interval, and muscles were collected 7 days following the second injury. To analyze tissue regeneration, multiple injuries experiments were performed using CTX and muscle were collected 14 days following the second injury. All techniques used are described in detail^35^.

### Histological and cells analyses

Muscles were weighed and frozen in liquid nitrogen-cooled isopentane as previously described^49^. Transverse cryosections (10 mm) were stained with haematoxylin and eosin. Collagen deposition was detected by Sirius Red staining^83^, fat tissue was stained by Oil Red O and hematoxylin^84^.

Transverse *TA* cryosection muscles were stained with LAMININ (Sigma) antibody to assess muscle fiber cross-sectional area (CSA) and number of muscle fiber. MHC isoforms were measured from cryosections obtained from the mid-belly of *TA* stained with MHC2b/BFF3, MHC1/BAD5 and MHC2a/SC71 antibodies (Hybridoma bank) as previously described^35^. Images were captured on a Zeiss AxioImagerZ1 microscope, and morphometric analysis was performed using MetaMorph7.5 (Molecular Devices). Entire muscle sections were analyzed from 3 to 5 animals per group. Sections and cultured cells were stained with antibodies for PW1, PAX7 (Developmental Studies HybridomaBank), KI67 (BD Biosciences and Abcam), MYOD (BD Biosciences and Santa Cruz), MF20 (Developmental Studies Hybridoma Bank), CASPASE3 (BD Biosciences) and PS6 (Cell signaling), species specific secondary antibodies coupled to AlexaFluor 488 (Molecular Probes), Cy3 or Cy5 (Jackson Immunoresearch), and nuclei were counterstained with DAPI (Sigma). For quantitative analysis, positive cells in at least 700 fibers from randomly chosen fields were counted from at least three animals per group.

### FACS analysis and primary culture

Hind-limb muscles were processed to obtain single cells as previously described^11,12^. In order to isolate satellite cells, cells were incubated with rat anti-mouse CD45-PE-Cy7 (eBiosciences), rat anti-mouse TER119-APC (Becton Dickinson), rat anti-mouse CD34-brilliant violet (Becton Dickinson), rant anti-mouse α7-integrin-A700, rat anti-mouse SCA1-FITC (eBiosciences). Satellite cells were isolated by α7-integrin^+^/CD34^+^/TER119^−^/CD45^−^/SCA1^−^. Primary antibodies were used at a concentration of 10 ng.ml-1. DAPI and 7AAD were used to collect live intact cells. To stain mitochondria we used 25 nM MitoTracker Red CMXRos (M7512). Flow cytometry was performed on a FACS Aria (Becton Dickinson) and for flow cyotmetry data analysis we used Flowjo software.

Cells were grown in high-glucose Dulbecco’s modified eagle medium (DMEM, Gibco) supplemented with 2.5 ng.ml-1 bFGF (Invitrogen), 20% heat-inactivated FBS (Invitrogen), 10% heat-inactivated horse serum (Gibco), 1% (v/v) penicillin-streptomycin (Gibco), 1% (v/v) LGlutamine (Gibco) and 1% (v/v) Na-pyruvate (Gibco). Medium was changed every 2 days. For clonal analysis, purified cell populations were grown on gelatin-coated dishes at low density for 5 days. For myogenic differentiation, three thousand satellite cells were seeded in 48-well plates for 1 week and transferred to differentiation medium (DM) for 2 days: DMEM containing 5% (v/v) horse serum and 1% (v/v) penicillinstreptomycin. To inhibit glycolysis proliferating satellite cells were treated for 24 h with 2-deoxiglucose (5 mM). To analyze the area of satellite cells, freshly sorted satellite cells were grown for 24 h and fixed with PFA 4%. Satellite cells area was measured using the ImageJ software. Proliferative capacity was quantified by counting at least 100–150 colonies, from at least three independent experiments. Fusion indexes were quantified by counting the number of nuclei in MF20+ cells per total number of nuclei^12,20^.

### Lactate assay

Three thousand purified satellite cells were seeded in 48-well plates. Lactate concentration was tested on cell culture medium 5 days after seeding. JM-K607–100 Lactate Assay Kit from cliniscience was used for the analysis.

### Allele-specific determination assays

To estimate the proportional allelic expression of *Pw1*, mouse hybrids of CAST/EiJ and C57BL/6 strains were used. RNA was isolated and cDNA generated from the hybrid 7–9 week old male mouse muscles, Allelic expression was estimated by pyrosequencing. Briefly, the cDNA was amplified by PCR in a reaction mixture (Bioline reagents, 21060) consisting of 3 µl of 10X reaction buffer, 0.6 µl of primer mix (10 µM forward and reverse primers), 0.9 µl of 50 mM MgCl_2_, 1.2 µl of dNTPs 2.5 mM, 0.125 µl of 5U/µl Taq polymerase, 23.175 µl of water and 1 µl of cDNA (diluted 20X). Reaction parameters were; 94 °C for 2 minutes, 40 cycles of 94 °C for 30 seconds, 60 °C for 30 seconds and 72 °C for 30 seconds, followed by 72 °C for 5 minutes. Amplification primers were F(biotinilated): AAGGCTCTGGTTGACAGTCGTG and R: TTCTCCTTGGTCTCACGGGC. The biotin conjugated forward strand of the amplicon was separated from the reverse strand and cleaned: The PCR mixture, 30 µl, was mixed with 1 µl of streptavidine sepharose beads (GE, 17-5113-01), 39 µl binding buffer (QIAGEN, 979006) and 10 µl water. The mixture was incubated on a shaker for 5 minutes and processed on a pyrosequencing vacuum station (QIAGEN, 9001529). The biotinilated DNA strands were captured (via their binding with streptavidin), washed in 70% ethanol, 0.2 M NaOH and 10 mM Tris-acetate (pH 7.6) for about 30 seconds each, the vacuum removed and the DNA deposited in a plate containing annealing buffer (QIAGEN, 979009) and a sequencing primer (AATGAAAGACTCCCCAC). The plate was incubated at 80 °C for three minutes before analysis on the pyrosequencer. Pyrosequencing was conducted using the PyroMark gold reagents (QIAGEN, 972812).

### RNA-Sequencing

Satellite cells were isolated by FACS from hindlimb muscle from 3 month *Pw1*^+/+^ and *Pw1*^m−/p−^ mice. We purified RNA from muscle stem cell population using RNAqueous-Micro total RNA isolation Kit (life-technologies) with a gDNA degradation step.

Directional libraries were prepared using Truseq Stranded mRNA sample preparation kit following the manufacturer’s instructions (Illumina). Libraries were checked for concentration and quality on DNA chips with the Bioanalyser Agilent (Illumina).

The libraries were quantified by fluorimetric measurements with the Qubit® dsDNA HS Assay Kit (ThermoFisher). 51-bp Single Read sequences were generated on the Hiseq2500 sequencer according to manufacturer’s instructions (Illumina). The multiplexing level was 2 samples per lane.

Reads were cleaned of adapter sequences and low-quality sequences using an in-house program (https://github.com/baj12/clean_ngs). Only sequences at least 25 nucleotides in length were considered for further analysis. Tophat version 1.4.1.1^85^, with default parameters, was used for alignment on the reference genome (GRCm38 from Ensembl database version 74). Genes were counted using HTSeq-count version 0.6.1^86^ (parameters: -t exon -i gene_id -m intersection-nonempty -s yes).The package used for the statistical analysis is DESeq2. For this analysis, a BH p-value adjustment was performed and the level of controlled false positive rate was set to 0.05. REVIGO software was used for the GO analysis.

## Electronic supplementary material

Supplementary Information (legends, supplementary figures)

Supplementary Table 1

Supplementary Table 2
