# Supplementary material for: The imprinted gene Pw1/Peg3 regulates skeletal muscle growth, satellite cell metabolic state, and self-renewal
Source: Sci Rep. 2018 Oct 2;8:14649. doi: 10.1038/s41598-018-32941-x (PMC6168517; doi:10.1038/s41598-018-32941-x)
Supplement: Supplementary file 1 — Supplementary Information (legends, supplementary figures) [file 41598_2018_32941_MOESM1_ESM.pdf]

Supplemental data

The imprinted gene *Pw1/Peg3* regulates skeletal muscle growth, satellite cell metabolic state, and self-renewal

Authors: Rosa Maria Correra[1], David Ollitrault[1,4], Mariana Valente[1,4], Alessia Mazzola[1], Bjorn T. Adalsteinsson[2], Anne C. Ferguson-Smith[3], Giovanna Marazzi[1,4,]*, David A. Sassoon[1,4,]*

Affiliations:

1. UMR S 1166 INSERM (Stem Cells and Regenerative Medicine Team), University of Pierre and Marie Curie Paris VI, Paris, 75634 and Institute of Cardiometabolism and Nutrition (ICAN), France
2. Department of Physiology Development and Neuroscience, Downing Street, University of Cambridge, United Kingdom
3. Department of Genetics, Downing Street, Cambridge, University of Cambridge, United Kingdom
4. (present address) Institut National de la Santé et de la Recherche Médicale (INSERM), Unit 970, Paris Cardiovascular Research Center, Université René Descartes Paris, Paris, France

Supplementary Figure legend

S1 Fig. Proportion of MHC isoforms is not impaired in *Pw1* null skeletal muscle.
(A) Histograms representing the percentages of MHC isoforms in 3 month $Pw1^{+/+}$ (+/+) and $Pw1^{m-/p-}$ (m-/p-) *TA* muscles (n=4 for each genotype). $Pw1^{m-/p-}$ *TA* muscles does not exhibit significant changes in slow (I) and fast (IIA-IIB) fibers proportions as compared to $Pw1^{+/+}$. **(B)** Cross-sections of $Pw1^{+/+}$, $Pw1^{+/p-}$, $Pw1^{m-/+}$, and $Pw1^{m-/p-}$ P0 hind limb muscle immunostained for PW1 (green) and LAMININ (orange). DAPI staining (blue) identifies nuclei. PW1 is not detectable in $Pw1^{+/p-}$ and $Pw1^{m-/p-}$ P0 muscles. Scale bar 20µm.
Values are expressed as mean ± s.e.m. In all the graphs statistical analyses were performed using Student's t-*test* $*P<0.05$, $**P<0.01$ *and* $***P<0.001$. In all the graphs $Pw1^{+/+}$ (+/+), $Pw1^{+/p-}$ (+/p-), $Pw1^{m-/+}$ (m-/+), and $Pw1^{m-/p-}$ (m-/p-).

S2 Fig. *Pw1* is expressed from the maternally inherited allele.
(A) *Pw1* allele specific expression in adult *TA* muscle before and five days after CTX injury from offspring of CAST/EiJ x CAST/EiJ (left-panel) and C57BL6/J x C57BL6/J (right-panel) crosses (n=3 for each genotype). Blue bar represent CAST/EiJ *Pw1* transcript and red bar represent the C57BL6/J *Pw1* transcript. **(B)** CAST/EiJ *Pw1* allele expression from ♀ CAST/EiJ x ♂ C57BL6/J cross and CAST/EiJ *Pw1* allele expression from ♀ C57BL6/J x ♂ C57BL6/J cross in adult *TA* muscle before and five days after CTX injury. CAST/EiJ *Pw1* allele expression from ♀ CAST/EiJ x ♂ C57BL6/J cross represent maternal *Pw1* transcript and CAST/EiJ *Pw1* allele expression from ♀ C57BL6/J x ♂ C57BL6/J cross represent the background of *Pw1* expression. **(C)** C57BL6/J *Pw1* allele expression from ♀ C57BL6/J x ♂ CAST/EiJ cross and C57BL6/J *Pw1* allele expression from ♀ CAST/EiJ x ♂ CAST/EiJ cross in adult *TA* muscle before and five days after CTX injury. C57BL6/J *Pw1* allele expression from ♀ C57BL6/J x ♂ CAST/EiJ cross represent maternal *Pw1* transcript and C57BL6/J *Pw1* allele expression from ♀ CAST/EiJ x ♂ CAST/EiJ cross represent the background of *Pw1* expression. The

background of *Pw1* expression is lower as compared to *Pw1* maternal allele expression from reciprocal hybrid offspring of C57BL6/J and CAST/EiJ mice.

Values are expressed as mean ± s.e.m. In all the graphs statistical analyses were performed using Student's t-test *P<0.05, **P<0.01 and ***P<0.001.

S3 Fig. Maternal *Pw1* is expressed at low levels in satellite cells and PICs.

(A) Expression levels of *Pw1* wildtype allele from real time PCR normalized to *Hprt1* gene expression in satellite cells (Qsat) and fibro adipogenic progenitors (FAPs) from uninjured adult *Pw1*$^{+/+}$ and *Pw1*$^{+/p-}$ hindlimb muscle (n=3 for each genotype). Maternal *Pw1* transcript is detected at low levels in satellite cells (Qsat) and fibro adipogenic progenitors (FAPs). In all the graphs *Pw1*$^{+/+}$ (+/+) and *Pw1*$^{+/p-}$ (+/p-).

S4 Fig. Regeneration in *Pw1* null mice after a single cardiotoxin injury appears normal.

(A) Representative photomicrographs of *TA* cross-sections two weeks after injury stained with hematoxylin and eosin **(upper panels)**, Oil-Red O **(middle panels)** and Sirius Red **(lower panels).** *Pw1*$^{m-/p-}$ muscle showed no differences in fat and fibrosis deposition as compared to *Pw1*$^{+/+}$. Scale bar=50µm. **(B)** Histograms showing the percentage of small (<800 µm2) and large (>3500 µm2) fibers after single CTX injury in *Pw1*$^{+/+}$ and *Pw1*$^{m-/p-}$ muscles (n= 3 for each assay and genotype). No significant differences in fiber size were found.

Values are expressed as mean ± s.e.m. In all the graphs statistical analyses were performed using Student's t-test *P<0.05, **P<0.01 and ***P<0.001. In all the graphs *Pw1*$^{+/+}$ (+/+) and *Pw1*$^{m-/p-}$ (m-/p-).

S5 Fig. *Pw1* deletion induces fiber hypertrophy and fat deposition after multiple muscle injuries

(**A**) Cross-sections of serially injured *TA* two weeks after second CTX injury stained with hematoxylin and eosin (upper panels), Oil-Red O (middle panels) and Sirius Red (lower panels). Adipocytes and fibrosis accumulates between regenerating fibers in *Pw1*$^{m-/p-}$ muscles as compared to *Pw1*$^{+/+}$ Scale bar = 50µm. (**B**) Quantification of fat

deposition in serially injured *TA* muscles two weeks after double CTX injury. Adipocytes infiltration was measured by Oil red O staining on muscle section and quantified as pixel values (n=5 for each assay and genotype). Fat deposition is increased in *Pw1*$^{m-/p-}$ muscles. (**C**) Histograms showing the percentage of small (<800 μm2) and large (>3500 μm2) fibers after single and double injury (n=5 for each assay and genotype). The percentage of small fibers after the second injury is significantly increased in *Pw1*$^{m-/p-}$ muscle as compared to the *Pw1*$^{+/+}$, while the percentage of large muscle fibers is significantly increased. (**D**) Histograms showing the percentage of fibers with centrally located nuclei in *Pw1*$^{+/+}$ and *Pw1*$^{m-/p-}$ *TA* two weeks after the second CTX injury (n=5 for each genotype). *Pw1*$^{m-/p-}$ displays a greater proportion of fibers with multiple central nuclei. (**E**) Histograms showing the number of *Pw1*$^{+/+}$ and *Pw1*$^{m-/p-}$ muscle fibers two weeks after single and double injury. *Pw1*$^{m-/p-}$ muscle shows a decrease in fiber number after single and double injury as compared to *Pw1*$^{+/+}$ muscles (n=3 for each assay and genotype). No differences in number of fibers between uninjured and injured *Pw1*$^{m-/p-}$ muscle were observed. (**F**) Quantification of PAX7 positive cells in *Pw1*$^{+/+}$ and *Pw1*$^{m-/p-}$ serially injured *TA* two weeks after the second injury. *Pw1*$^{m-/p-}$ muscle showed a decline of PAX7 positive cells (n=3 for each assay and genotype). Values represent the mean number of positive cells ± s.e.m. per 100 fibers.

In all the graphs values are expressed as mean ± s.e.m. NI=non injured; SI=single injured; DI=double injured. Statistical analyses were performed using Student's t-test *P<0.05, **P<0.01 and ***P<0.001. In all the graphs *Pw1*$^{+/+}$ (+/+) and *Pw1*$^{m-/p-}$ (m-/p-).

S6Fig. *Pw1* null satellite cells express altered levels of genes involved in mitochondrial function.

(**A**) Validation of RNA-seq result. Quantification of mRNA expression levels of genes involved in mitochondrial organization (*Gabarapl1, Ppif, Akt3, Atp2a, Vps13c, Mgarp, Hsp1l*) from real time PCR analysis of satellite cells from in 3 month *Pw1*$^{+/+}$ and *Pw1*$^{m-/p-}$ (n=3 for each genotype). Values were normalized to *Tbp*. The mRNA levels of the analyzed genes are significantly lower in *Pw1*$^{m-/p-}$ satellite cells. (**B**) Histogram represents FSC-A MFIs of *Pw1*$^{m-/p-}$ satellite cells relative to *Pw1*$^{+/+}$. Loss

of *Pw1* has no effect on satellite cell size (n=6 for each genotype). (**C**) Histograms showing proportions of PAX7+/PS6- (G0 satellite cells, blue) and PAX7+/PS6+ (G_{Alert} satellite cells, red) from 3 month *Pw1*$^{+/+}$ and *Pw1*$^{m-/p-}$ *TA*. *Pw1* null satellite cells are not in a G_{Alert} state in resting adult muscle (n=4 for each genotype).

Value are expressed as mean ± s.e.m.. In all the graphs statistical analyses were performed using Student's t−test *P < 0.05, **P < 0.01 and ***P < 0.001. In all the graphs *Pw1*$^{+/+}$ (+/+) and *Pw1*$^{m-/p-}$ (m-/p-).

S Fig1

A

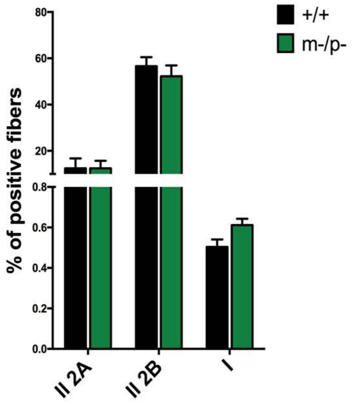

+/+
m-/p-
% of positive fibers
80
60
40
20
0.8
0.6
0.4
0.2
0.0
II 2A
II 2B
I

B

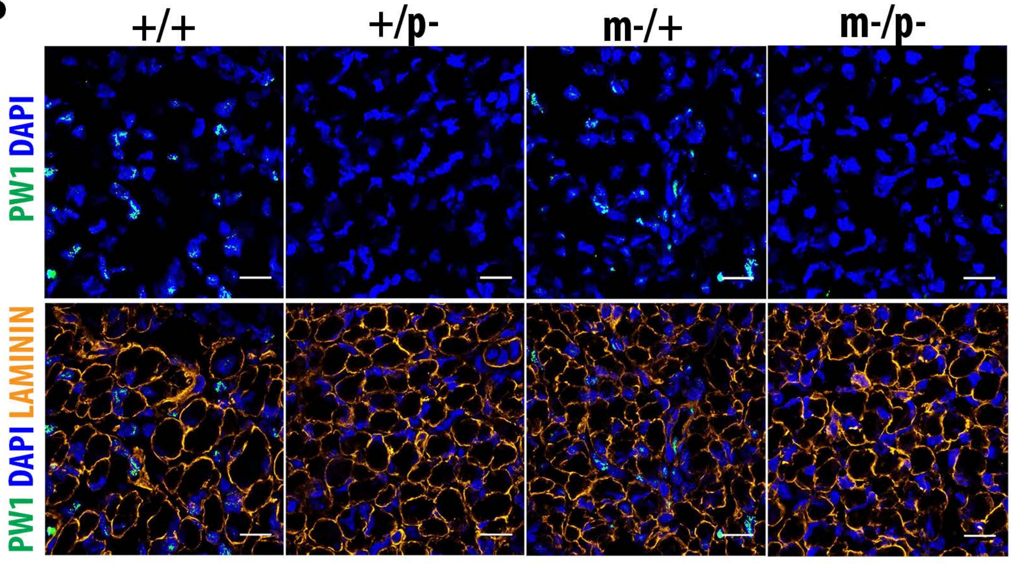

+/+
+/p-
m-/+
m-/p-
PW1 DAPI
PW1 DAPI LAMININ

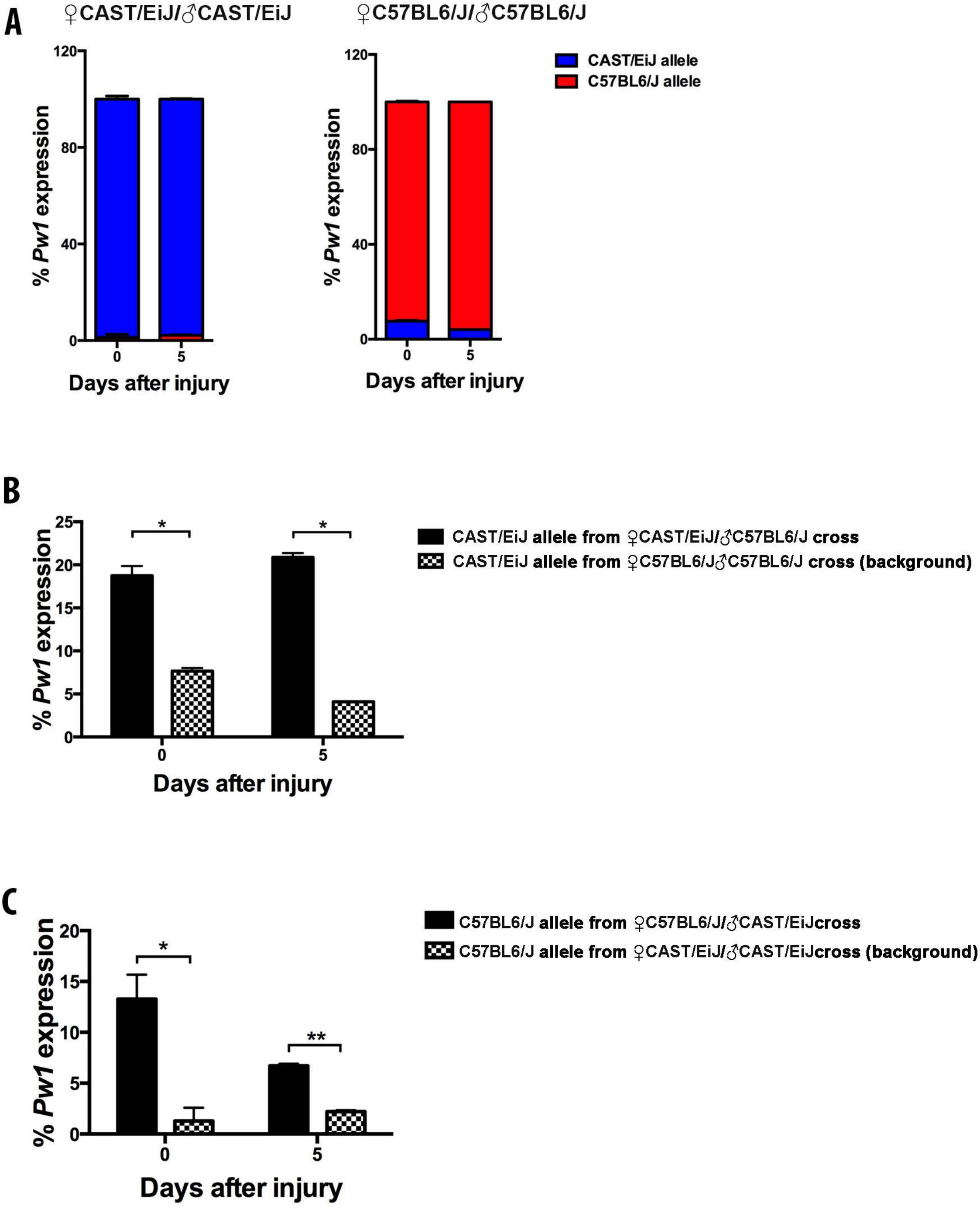
A
♀CAST/EiJ/♂CAST/EiJ
♀C57BL6/J/♂C57BL6/J
CAST/EiJ allele
C57BL6/J allele
% Pw1 expression
Days after injury
B
CAST/EiJ allele from ♀CAST/EiJ/♂C57BL6/J cross
CAST/EiJ allele from ♀C57BL6/J♂C57BL6/J cross (background)
% Pw1 expression
Days after injury
C
C57BL6/J allele from ♀C57BL6/J/♂CAST/EiJcross
C57BL6/J allele from ♀CAST/EiJ/♂CAST/EiJcross (background)
% Pw1 expression
Days after injury

Sup Fig 3

A

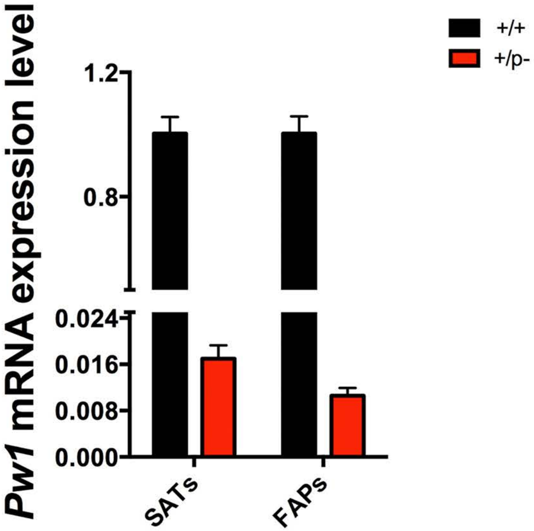
+/+
+/p-
1.2
0.8
0.024
0.016
0.008
0.000
Pw1 mRNA expression level
SATs
FAPs

Sup Fig 4

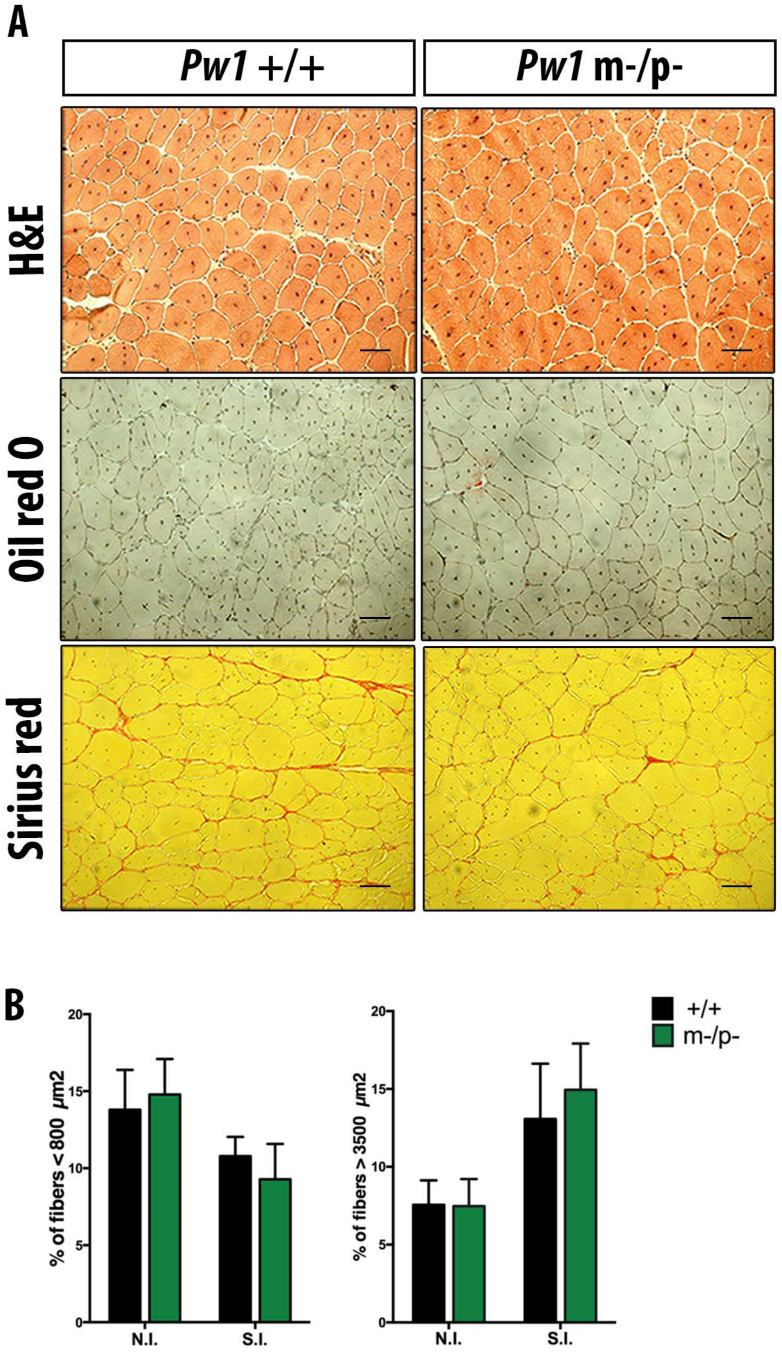
A
Pw1 +/+
Pw1 m-/p-
H&E
Oil red O
Sirius red
B
% of fibers <800 μm2
% of fibers > 3500 μm2
0
5
10
15
20
N.I.
S.I.
+/+
m-/p-

Sup Fig 5

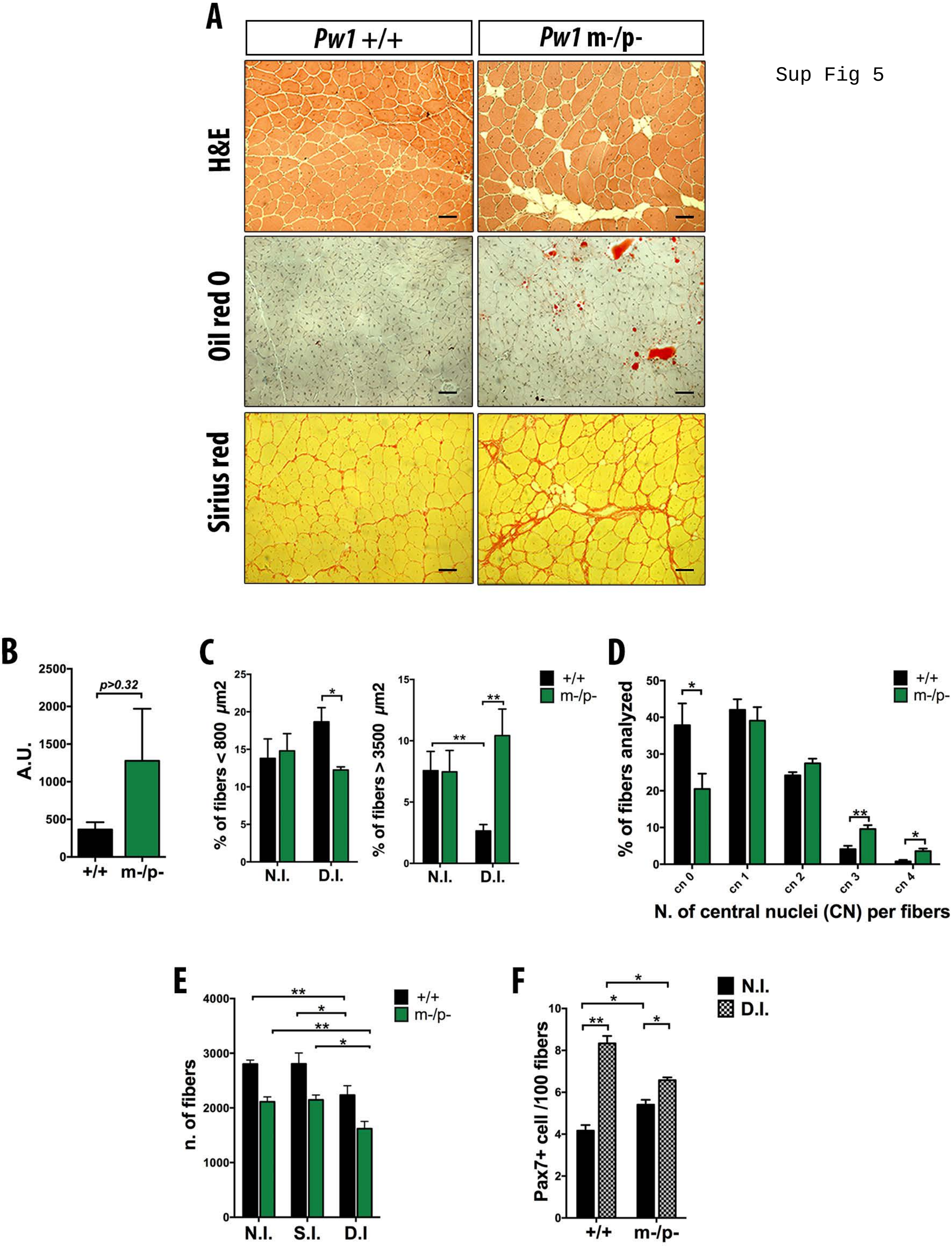
A
Pw1 +/+
Pw1 m-/p-
H&E
Oil red O
Sirius red
B
p>0.32
A.U.
+/+
m-/p-
C
% of fibers < 800 μm2
% of fibers > 3500 μm2
N.I.
D.I.
+/+
m-/p-
D
% of fibers analyzed
cn 0
cn 1
cn 2
cn 3
cn 4
N. of central nuclei (CN) per fibers
E
n. of fibers
N.I.
S.I.
D.I
F
Pax7+ cell /100 fibers
N.I.
D.I.

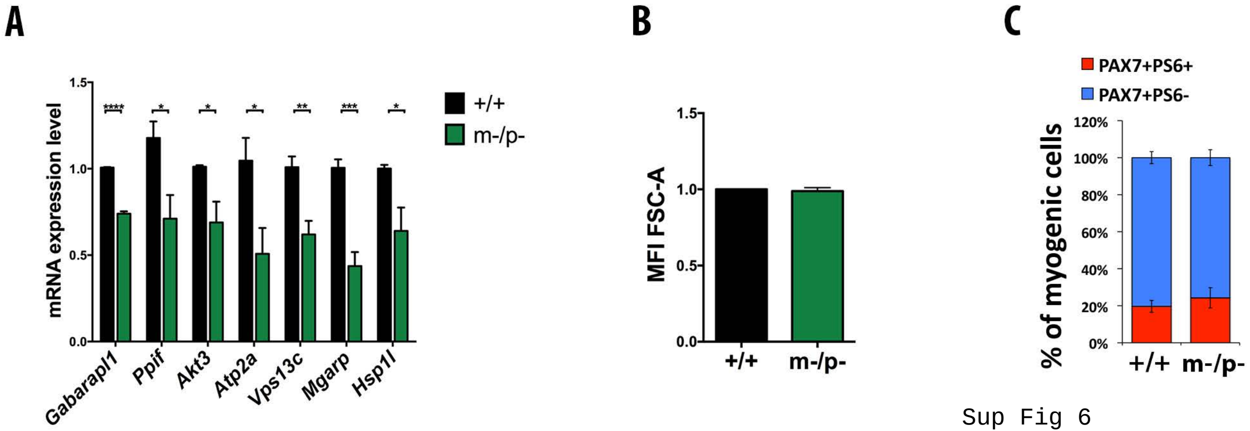
A
B
C
mRNA expression level
1.5
1.0
0.5
0.0
****
*
*
*
**
***
*
Gabarapl1
Ppif
Akt3
Atp2a
Vps13c
Mgarp
Hsp1l
+/+
m-/p-
MFI FSC-A
1.5
1.0
0.5
0.0
+/+
m-/p-
PAX7+PS6+
PAX7+PS6-
% of myogenic cells
120%
100%
80%
60%
40%
20%
0%
+/+
m-/p-

Sup Fig 6

Supplemental Tables

The imprinted gene *Pw1/Peg3* regulates skeletal muscle growth, satellite cell metabolic state, and self-renewal

Authors: Rosa Maria Correra[1], David Ollitrault[1,4], Mariana Valente[1,4], Alessia Mazzola[1], Bjorn T. Adalsteinsson[2], Anne C. Ferguson-Smith[3], Giovanna Marazzi[1,4,]*, David A. Sassoon[1,4,]*

Affiliations:

1. UMR S 1166 INSERM (Stem Cells and Regenerative Medicine Team), University of Pierre and Marie Curie Paris VI, Paris, 75634 and Institute of Cardiometabolism and Nutrition (ICAN), France
2. Department of Physiology Development and Neuroscience, Downing Street, University of Cambridge, United Kingdom
3. Department of Genetics, Downing Street, Cambridge, University of Cambridge, United Kingdom
4. (present address) Institut National de la Santé et de la Recherche Médicale (INSERM), Unit 970, Paris Cardiovascular Research Center, Université René Descartes Paris, Paris, France

Table S1. RNA-seq analysis of differentially expressed genes in *Pw1* mutant and wildtype satellite cells.

Table S2. Gene ontology analysis of genes downregulated and upregulated in *Pw1* mutant and wildtype satellite cells. GO terms between the downregulated gene set associated with mitochondrial function and cell death are highlighted in red.
